# *Acanthosaura syrax* sp. nov., a new species of mountain horned dragon from high-elevation habitats of western Thailand (Squamata, Agamidae)

**DOI:** 10.3897/zookeys.1287.186636

**Published:** 2026-07-31

**Authors:** Poramad Trivalairat, Krittiya Trivalairat, Nawee Moungkhaek, Simon Caponov, Benchapol Lorsunyaluck

**Affiliations:** 1 Princess Agrarajakumari Faculty of Nursing, Chulabhorn Royal Academy, 906 Thung Song Hong, Lak Si, Bangkok, 10210, Thailand Department of Microbiology, Faculty of Science, Mahidol University Bangkok Thailand; 2 Laboratory for the Exploration of Ectoparasites and Carnivorous Hirudineans (L.E.E.C.H.), Department of Zoology, Faculty of Science, Kasetsart University, 50 Ngam Wong Wan Road, Chatuchak, Bangkok, 10900, Thailand Princess Agrarajakumari Faculty of Nursing, Chulabhorn Royal Academy Bangkok Thailand; 3 Department of Microbiology, Faculty of Science, Mahidol University, 272 Rama VI Rd, Thung Phaya Thai, Ratchathewi, Bangkok, 10400, Thailand Akkhraratchakumari Veterinary College, Walailak University Nakhon Sithammarat Thailand; 4 Mae Wong National Park, Pang Ta Wai, Pang Sila Thong District, Kamphaeng Phet, 62120, Thailand Laboratory for the Exploration of Ectoparasites and Carnivorous Hirudineans (L.E.E.C.H.), Department of Zoology, Faculty of Science, Kasetsart University Bangkok Thailand; 5 Panalai Veterinary Hospital, 100/5 Tiwanon Rd, Pak Kret, Nonthaburi, 11120, Thailand Mae Wong National Park Kamphaeng Phet Thailand; 6 Akkhraratchakumari Veterinary College, Walailak University, 222 Thaiburi, Thasala, Nakhon Sithammarat, 80160, Thailand Panalai Veterinary Hospital Nonthaburi Thailand

**Keywords:** COI, Mae Wong National Park, morphology, ND2, reptile, Tak–Kamphaeng Phet Province, taxonomy, tropical rainforest

## Abstract

A new species of mountain horned dragon, *Acanthosaura syrax***sp. nov**., is described from the high–elevation evergreen forests of Mae Wong National Park in western Thailand. Morphological comparisons distinguish the new taxon from its congeners by its smaller body size and the fact that it possesses the smallest nuchal and dorsal scales recorded within the genus. Phylogenetic analysis of mitochondrial ND2 and COI gene sequences further supports this distinction, identifying the new species as a reciprocal monophyletic lineage with 76% and 99% nodal support in maximum–likelihood analyses and 0.78 and 1.00 nodal support in Bayesian Inference analyses based on ND2 and COI genes, respectively. Interspecific pairwise genetic distances between *A. syrax***sp. nov**. and other congeners range from 14.1–15.9% in ND2 gene and 14.96–28.13% in COI gene, while intraspecific variation remains low at 0–0.01% and 0–0.34% in ND2 and COI, respectively. This discovery brings the number of *Acanthosaura* species to 23 and highlights the significant and unique biodiversity found within the high–altitude evergreen ecosystems of western Thailand.

## Introduction

*Acanthosaura* Gray, 1831, commonly known as mountain horned dragons or pricklenape lizards, is a genus of agamid lizards characterized by a distinctive row of nuchal and dorsal crests (Boulenger 1912; Taylor 1963; Das 2010; Trivalairat et al. 2020, 2022). The genus includes a diverse assemblage of species distributed across Southeast Asia, ranging from Myanmar through Thailand, Cambodia, Laos, Vietnam, and southern China, extending into the Malaysian Peninsula and archipelagoes (Gray 1831; Taylor 1963; Grismer et al. 2008; Manthey 2008; Das 2010; Wood Jr et al. 2010; Ananjeva et al. 2011, 2020; Pauwels et al. 2015; Mulcahy et al. 2018; Liu and Rao 2019; Nguyen et al. 2019; Liu et al. 2020, 2022; Trivalairat et al. 2020, 2022; Uetz et al. 2024, 2026; Le et al. 2025; Ngo et al. 2025). Molecular and morphological data indicated the presence of 22 described *Acanthosaura* species, with some taxa being endemic and recently described within the last decade (Nguyen et al. 2019; Ananjeva et al. 2020; Liu et al. 2020, 2022; Trivalairat et al. 2020, 2022; Uetz et al. 2024; Le et al. 2025; Ngo et al. 2025).

In Thailand, the known diversity of *Acanthosaura* taxa has increased in recent years, currently comprising at least seven species: *Acanthosaura armata* (Gray, 1827) from the southern region; *A. aurantiacrista* Trivalairat, Kunya, Chanhome, Sumontha, Vasaruchapong, Chomngam & Chiangkul, 2020 from the north-western region; *A. cardamomensis* Wood, Grismer, Grismer, Neang, Chav & Holden, 2010 from the eastern region; *A. crucigera* Boulenger, 1885 from the western region; *A. lepidogaster* (Cuvier, 1829) from the northern region; *A. phuketensis* Pauwels, Sumontha, Kunya, Nitikul, Samphanthamit, Wood & Grismer, 2015 from Phuket Island and the south-western region; *A. meridiona* Trivalairat, Sumontha, Kunya & Trivalairat, 2022 from the southern region; and *A. rubrilabris* Liu, Rao, Hou, Orlov, Ananjeva & Zhang, 2022 from the northern region (Thai National Parks 2013b); Uetz et al. 2026). Some of them, such as *A. lepidogaster* and *A. crucigera*, have overlapping distributions within the Tenasserim Mountain range, extending across western Thailand and into southern Myanmar (Hardwicke and Gray 1827; Cuvier 1829; Boulenger 1885; Pauwels et al. 2003; Grismer et al. 2008; Wood Jr et al. 2010; Pauwels et al. 2015; Trivalairat et al. 2020, 2022). However, within Tenasserim Mountain range, there is a potential for discovering additional, yet undescribed, species of *Acanthosaura* in Thailand from unsurveyed areas.

Mae Wong National Park, Tak–Kamphaeng Phet Province, Thailand is an area characterized by high-elevation evergreen forests reaching up to 1,964 m a.s.l., with a cold temperate climate year-round (Thai National Parks 2013a). This area is reported as a covered distribution range of *A. lepidogaster* (Thai National Parks 2013a). During field surveys, none of them were encountered, whereas specimens *Acanthosaura* collected from this site exhibited a distinct morphotype, notably with the smallest nuchal and dorsal crests scales observed in the genus. Based on morphological and molecular analyses, these individuals represent a previously unrecognized species of *Acanthosaura*, herein described as the twenty-third species in the genus.

## Materials and methods

### Specimen sampling and preparation

Specimens were conducted within range of Mae Wong National Park, Tak–Kamphaeng Phet Province, Thailand (16°06'01.6"N, 99°06'28.0"E) by hand. Photographs were taken to document the coloration pattern in life prior to euthanasia. Euthanasia was performed using a freezing technique at –10 °C for one week, at this species can hibernate in low-temperature conditions for a few days. The specimens were fixed in 10% neutral buffered formalin (NBF) for one week to preserve tissues. Following fixation, they were rinsed with distilled water and transferred to 70% ethanol for storage.

For molecular analyses, fresh liver samples were dissected into small pieces and stored in absolute ethanol. All specimens were deposited at Natural History Museum, National Science Museum, Technopolis, Pathum Thani Province, Thailand (**THNHM**).

### Morphological characteristics

Meristic and measured morphological characters were noted on the left side followed Pauwels et al. (2015), Liu et al. (2020), Ananjeva et al. (2020), and Ngo et al. (2025). Measurements were performed with vernier calipers to the nearest 0.01 mm. Fifty-one morphometric and meristic data were collected following: **SVL** – snout–vent length, measured from the tip of the snout to the tip of the vent; **TaL** – tail length, measured from the posterior margin of the vent to the tip of the tail; **TBW** – tail base width, maximum width at tail base; **HL** – head length, measured from the posterior edge of the rectis of the jaw to the tip of the snout; **HW** – head width, maximum head width, the width at the level of the tympanum; **HD** – maximum head height, measured across the parietal region; **SL** – snout length, measured from the anterior edge of the orbit to the tip of the snout; **ORBIT** – orbit diameter, measured from the posterior to the anterior edge of the orbit; **EYE** – eye diameter, measured from the posterior to the anterior edge of the eye; **TD** – tympanum diameter, measured horizontally from the anterior to the posterior border of the tympanum; **TN** – scales absent on tympanum (0) or present (1); **PS** – postorbital spine length, measured from the base to the tip of the spine; **NSL** – maximum length of the largest spine in the nuchal crest measured from the base to the tip, previously SNL; **NN** – number of the nuchal crest; **DS** – maximum length of the largest spine in the dorsal crest measured from the base to the; **NSW** – maximum width of the spines in the nuchal crest, measured at the base, previously used as WNC; **DIAS** – length of the diastema, measured from the posterior end of the nuchal crest to the anterior end of the dorsal crest; **DIASN** – number of scales in the vertebral crest scale diastema counted from the posterior end of the nuchal crest to the anterior end of the dorsal crest; **FOREL** – forelimb length, measured from axilla to the proximal edge of the palmar region; **HINDL** – hindlimb length, measured from the groin to the proximal edge of the plantar region; **SUPRAL** – number of supralabials; **INFRAL** – number of infralabials; **VENT** – number of ventral scales counted at the midline from the anterior edge of the shoulders to the edge of the vent; **FI** – number of subdigital lamellae on the fourth finger; **TO** – subdigital lamellae on the fourth toe; **OS** – length of the occipital spine, measured from the base to the tip; **NSSOS** – number of scales surrounding the occipital spine; **CS** – number of canthus rostralis–supraciliary scales, counted from the nasal scale to the posterior end of the ridge at the posterior margin of the orbit; **RW** – rostral width; **RH** – rostral height; **RS** – number of scales bordering the rostral scale; **NS** – number of scales between the nasals; **NCS** – number of scales between the fifth canthals; **NSCSL** – number of scales from the fifth canthal to the fifth supralabial; **NR** – number of scales between the nasal and the rostral scales; **NSSLC** – number of scales between the seventh supralabial and the sixth canthal; **MW** – mental width; **MH** – mental height; **PM** – number of scales bordering the mental; **YAS** – presence (1) or absence (0) of a **Y**-shaped arrangement of enlarged scales on the snout; **ND** – presence (1) or absence (0) of a black, diamond shaped, nuchal collar; **LKP** – presence (1) or absence (0) of a pale knee patch; **BEP** – presence (1) or absence (0) of a black eye patch; **ESBO** – presence (1) or absence (0) of elliptical scales below the orbit; **GP** – size of gular pouch scored as absent, small, medium or large; **OF** – presence (1) or absence (0) of oblique fold anterior to the forelimb insertion; **NEBO** – Number of elliptical scales below the orbit; **NSSOB** – Number of scales surrounding the orbital spine; **PL** – penis length; **PW** – penis width, measured at the tip; **EGG** – number of eggs per clutch. Additionally, the length of all nuchal scales (**NSL1**–**NSLn**, where “**n**” corresponds to the total number of nuchal scales) and the length of the first ten dorsal (**DS1**–**DS10**) were measured. Comparative materials of all currently recognised species of *Acanthosaura* in Thailand were examined from the Queen Saovabha Memorial Institute, Thai Red Cross Society, Bangkok Province, Thailand (**QSMI**) and the Natural History Museum, National Science Museum, Technopolis, Pathum Thani Province (**THNHM**).

### Molecular analyses

Molecular data were generated from specimens collected in the Mae Wong National Park Range. To establish the phylogenetic context, these data were compared against sequences for various *Acanthosaura* taxa and a designated outgroup, *Calotes emma* Gray, 1845, which were retrieved from the GenBank database Appendix 1: Table A1) (Macey et al. 1997a, 2000; Zug et al. 2006; Okajima and Kumazawa 2010; Wood Jr et al. 2010; Pauwels et al. 2015; Yu et al. 2015; Liu and Rao 2019; Nguyen et al. 2019; Liu et al. 2020, 2022; Trivalairat et al. 2020, 2022; Uetz et al. 2024; Le et al. 2025; Ngo et al. 2025). Liver tissue samples were used for the extraction of genomic DNA. The extraction process was carried out using a TIANamp Genomic DNA Kit. Tissue samples were first subjected to lysis by incubation with proteinase K for 3–4 h at a temperature of 56 °C. Following lysis, the purified DNA was recovered from the spin column by elution with 200 μl of buffer.

A 698-base pair (bp) fragment of the NADH dehydrogenase subunit 2 (ND2) gene and 600–bp fragment of the Cytochrome c oxidase subunit I (COI) gene were amplified via Polymerase Chain Reaction (PCR) using EP0402 TAQ DNA POLYMERASE. The following primer pair was used for amplification: METF6 (L4437a; 5’–AAG CTT TCG GGC CCA TAC C–3’) and ACANTHND2.833. R1 (5’–AGG GAG GTT ATT GTT GCT AG–3’) for ND2; Chmf4 (5’–TYT CWA CWA AYC AYA AAG AYA TCG G–3’) and Chmr4 (5’–ACY TCR GGR TGR CCR AAR AAT CA–3’) for COI (Macey et al. 1997b; Kalyabina-Hauf et al. 2004; Gamble et al. 2008; Wood Jr et al. 2010; Che et al. 2012).

The thermal cycling protocol for the ND2 gene amplification was performed for 32 cycles as follows (Jackman et al. 2008): initial denaturation for 2 min at 95 °C, followed by 95 °C for 35 s, annealing at 50 °C for 35 s and extension at 72 °C for 154 s per cycle (Jackman et al. 2008). Additionally, the thermal cycling protocol for the COI gene amplification was performed over 32 cycles as follows (Jackman et al. 2008): initial denaturation for 5 min at 94 °C, followed by 35 cycles at 94 °C for 45 s, 72 °C for 45 seconds, and 72 °C for 7 minutes (Che et al. 2012). The resulting PCR products were submitted to Macrogen Co. in South Korea for sequencing. The successfully obtained sequences have been deposited in the GenBank repository, with the corresponding accession numbers detailed in Appendix 1: Table A1.

### Phylogenetic analysis

The DNA sequences were managed and prepared for phylogenetic analysis. Sequence editing and alignment were performed using the Clustal Omega (accessible at https://www.ebi.ac.uk/Tools/msa/clustalo/), implemented through the MEGA6 software suite (Tamura et al. 2013). All alignment parameters were set to default values. To ensure the integrity of the coding region, all sequences were translated into amino acids to verify the absence of frameshift mutations or premature stop codons. Following sequence alignment, pairwise genetic distances were calculated both within and between mitochondrial clades using MEGA6.

The phylogenetic reconstructions were conducted using two complementary approaches: Maximum Likelihood (ML) and Bayesian Inference (BI). The optimal models of molecular evolution for both datasets were selected using ModelFinder (Kalyaanamoorthy et al. 2017) implemented in IQ-TREE, based on the Bayesian Information Criterion (BIC). The GTR+I+G substitution model was identified as the best-fit model for both the ND2 and COI datasets and was subsequently applied in both analytical frameworks.

The ML analysis was performed using IQ-TREE v. 2.2.2.6 (Minh et al. 2020), and nodal support was evaluated using 1,000 ultrafast bootstrap replicates (Hoang et al. 2018). Bayesian Inference was conducted using MrBayes v. 3.2.7 (Ronquist et al. 2012) by setting the corresponding parameters for the GTR+I+G model (*nst* = 6, *rates* = invgamma). The Markov Chain Monte Carlo (MCMC) analysis was run for 1,000,000 generations across two independent runs with four chains each, sampling trees every 1,000 generations. To ensure convergence, the first 25% of the sampled trees were discarded as burn-in, and the remaining trees were used to generate a consensus tree and calculate posterior probabilities.

Clade support was considered statistically significant when ultrafast bootstrap (UFB) values were ≥ 95% (Felsenstein 2004; Minh et al. 2020) and Bayesian posterior probabilities (BPP) were ≥ 0.95 (Huelsenbeck and Ronquist 2001).

## Results

### Species description

#### Acanthosaura

Taxon classification

Animalia

SquamataAgamidae

Gray, 1831

222B1EF6-96E8-5FB5-999A-8C2715595FBC

##### Type species.

*Acanthosaura armata* (Gray, 1827).

##### Diagnosis.

Body somewhat compressed, usually with a rather sharp dorsal ridge and a dorsal crest larger in males; dorsal scales unequal, heterogenous; a gular pouch may be present; a fold cross shoulder usually conspicuous; tympanum naked or partially covered by scales; tail somewhat flattened, the scales keeled and larger than dorsal body scales, longer than broad; no femoral or preanal pores (Gray 1831; Taylor 1963).

##### Distribution.

*Acanthosaura* species can be found across Southeast Asia and extending southward to Malay Peninsula (Table 1).

**Table 1.** Distribution of *Acanthosaura* species.

**Country**	***Acanthosaura* species**	**Reference**
Cambodia	*A. capra*, *A. cardamomensis*, *A. coronata*, *A. crucigera*;	Günther (1861); Boulenger (1885); Wood Jr et al. (2010); Uetz et al. (2026)
China (southern region)	*A. armata*, *A. lepidogaster*, *A. liui*, *A. longicaudata*, *A. rubrilabris*, *A. tongbiguanensis*	Gray (1827); Cuvier (1829); Taylor (1963); Das (2010); Liu and Rao (2019); Liu et al. (2020, 2022); Uetz et al. (2026)
Laos	*A. coronata*, *A. lepidogaster*, *A. nataliae*, *A. prasina*	Cuvier (1829); Günther (1861); Orlov et al. (2006); Ananjeva et al. (2020); Uetz et al. (2026)
Malay Peninsula	*A. armata*, *A. bintangensis*, *A. titiwangsaensis*	Gray (1827); Wood Jr et al. (2009); Das (2010); Uetz et al. (2026)
Myanmar	*A. armata*, *A. crucigera*, *A. lepidogaster*	Gray (1827); Cuvier (1829); Boulenger (1885); Taylor (1963); Das (2010); Uetz et al. (2026)
Thailand	*A. armata*, *A. aurantiacrista*, *A. cardamomensis*, *A. crucigera*, *A. lepidogaster*, *A. meridiona*, *A. phuketensis*, *A. rubrilabris*	Gray (1827); Cuvier (1829); Boulenger (1885); Taylor (1963); Das (2010); Wood Jr et al. (2010); Thai National Parks (2013b); Pauwels et al. (2015); Trivalairat et al. (2020, 2022); Liu et al. (2022); Uetz et al. (2026)
Vietnam	*A. brachypoda*, *A. capra*, *A. cardamomensis*, *A. coronata*, *A. cuongi*, *A. grismeri*, *A. lepidogaster*, *A. murphyi*, *A. nataliae*, *A. phongdienensis*, *A. prasina*	Cuvier (1829); Günther (1861); Orlov et al. (2006); Wood Jr et al. (2010); Ananjeva et al. (2011); Nguyen et al. (2018, 2019); Ananjeva et al. (2020); Le et al. (2025); Ngo et al. (2025); Uetz et al. (2026)

#### Acanthosaura syrax sp. nov.

Taxon classification

Animalia

SquamataAgamidae

A3F1550F-96B2-5117-A0EC-7F5064E02E92

https://zoobank.org/7C2AC36D-53CF-4DB4-91B3-40FC80918F8C

Figs 1, 2, 3, 4, 5

##### Material examined.

**Figure 1.**
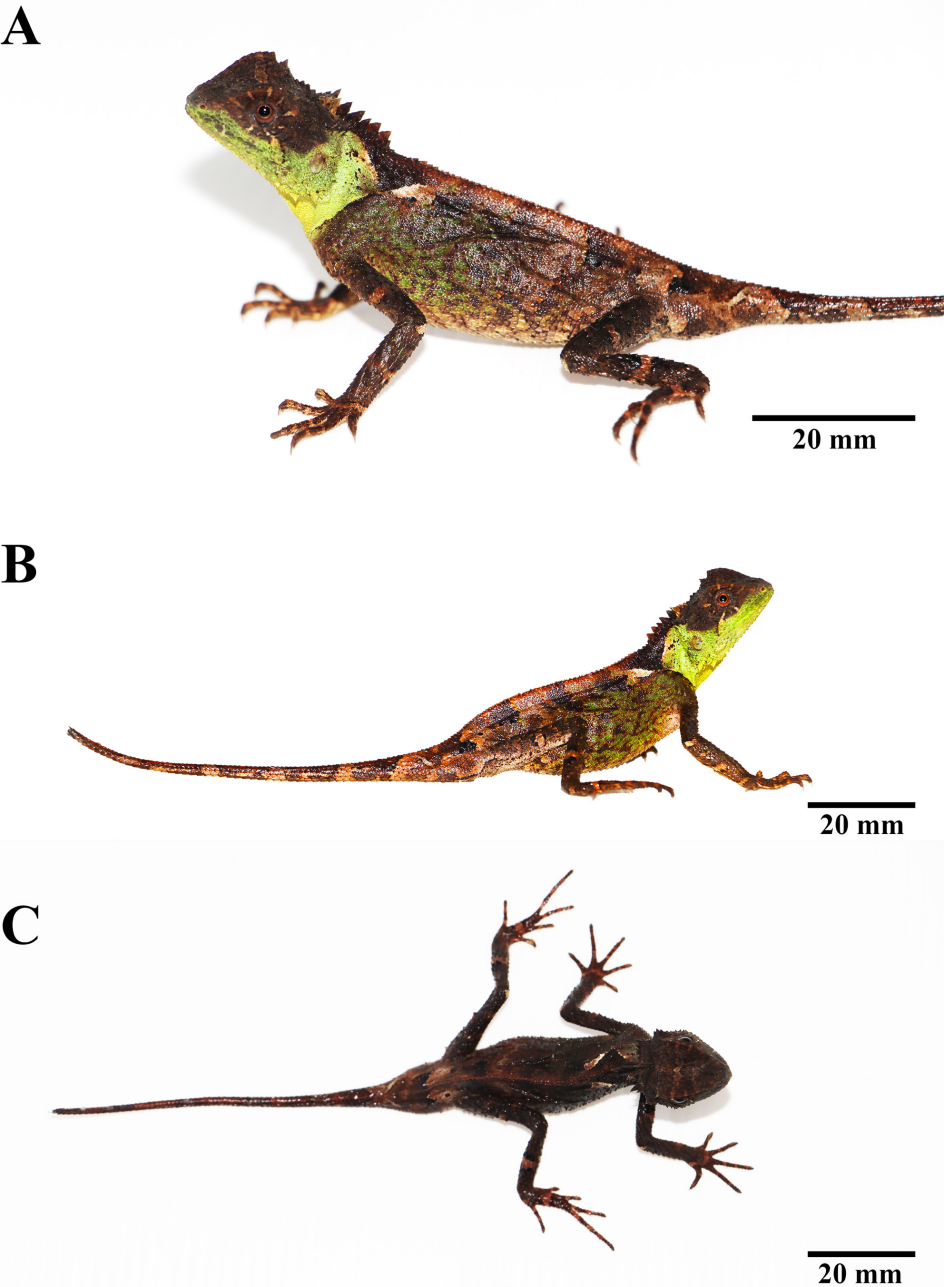
Adult male holotype (THNHM31129) of *Acanthosaura syrax* sp. nov. from Mae Wong District, Kampheang Phet Province, Thailand (16°05'43.5"N, 99°07'25.2"E), at 1,340 m a.s.l. **A**. Left lateral view; **B**. Right lateral view; **C**. Dorsal view.

**Figure 2.**
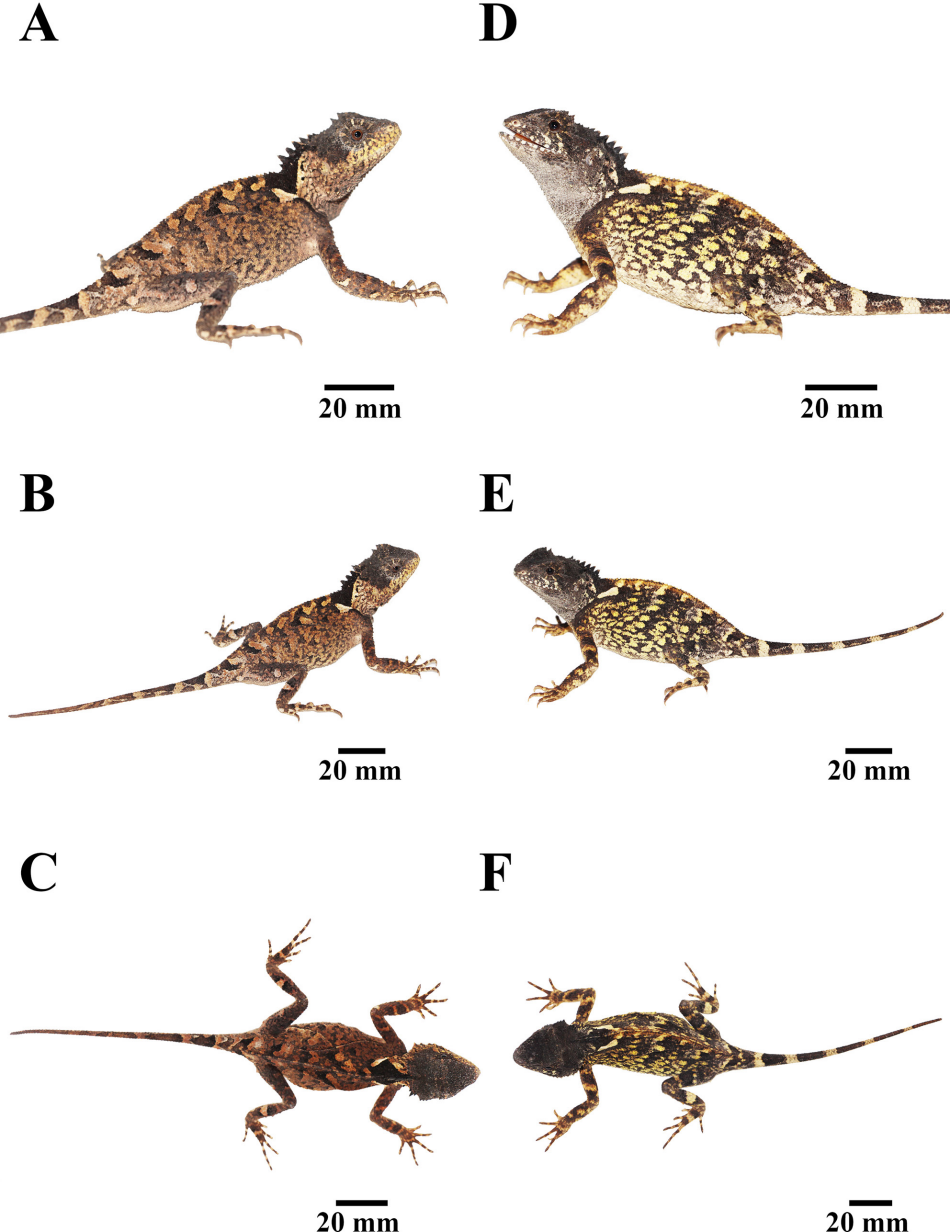
Adult female paratype (THNHM31130 for (**A–C**), THNHM31131 for (**D–F**)) of *Acanthosaura syrax* sp. nov. **A, B**. Right lateral view; **C, F**. Dorsal view. **D, E**. Left lateral view.

**Figure 3.**
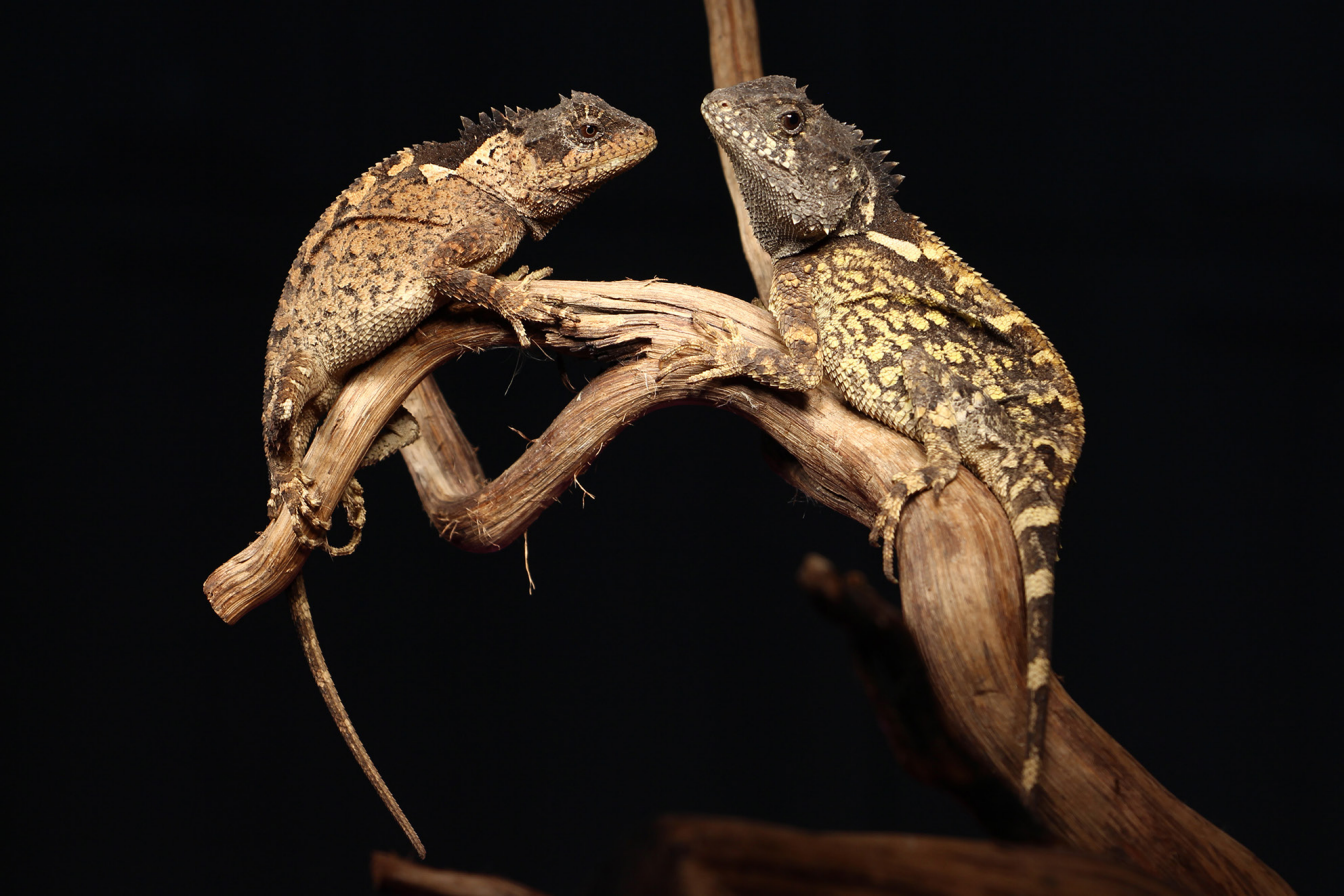
Adult female paratypes of *Acanthosaura syrax* sp. nov. (left for THNHM31130 and right for THNHM31131).

**Figure 4.**
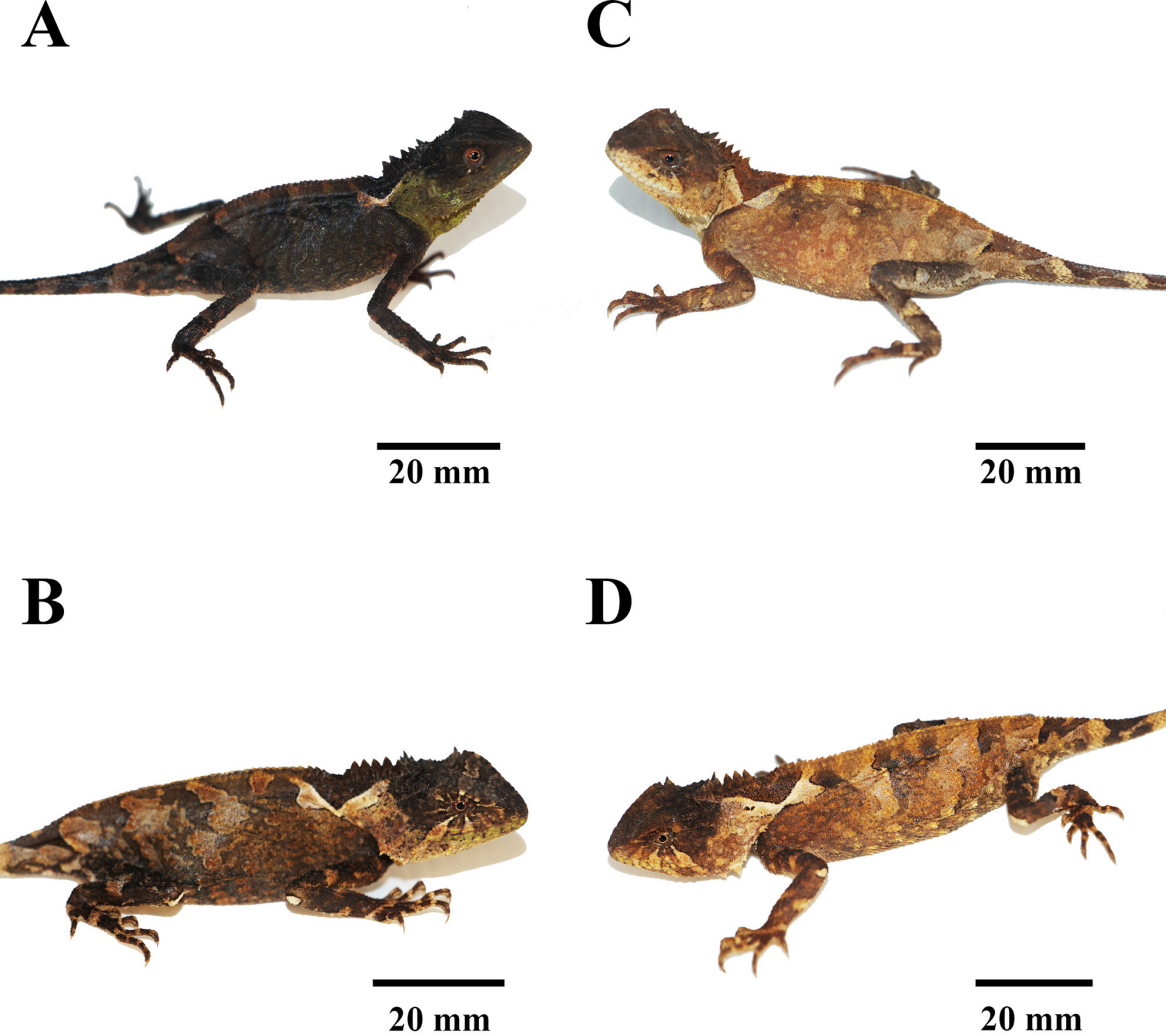
Paratype specimens of *Acanthosaura syrax* sp. nov. **A**. Adult male (THNHM331135); **B**. Adult female (THNHM31132); **C**. Adult female (THNHM31133); **D**. Adult female (THNHM31134).

***Holotype***: Thailand • 1 mature male; Kamphaeng Phet Province, Mae Wong District, Mae Wong National Park, Khun Nam Yen; 16°05'43.5"N, 99°07'25.2"E; 1,340 m a.s.l.; 23 Apr 2025; collected by Poramad Trivalairat & Krittiya Trivalairat; THNHM31129 (Fig. 1). ***Paratypes***: Thailand • 2 mature females; same data as holotype; THNHM31130–31131; • 3 mature females, 2 mature males; same locality as holotype; 26 May 2025; collected by Poramad Trivalairat & Krittiya Trivalairat; THNHM31132–31136 (Figs 2, 3, 4). All collected specimens were kept in 70% alcohol and deposited at the Natural History Museum, National Science Museum, Technopolis, Pathum Thani Province, Thailand (THNHM).

**Figure 5.**
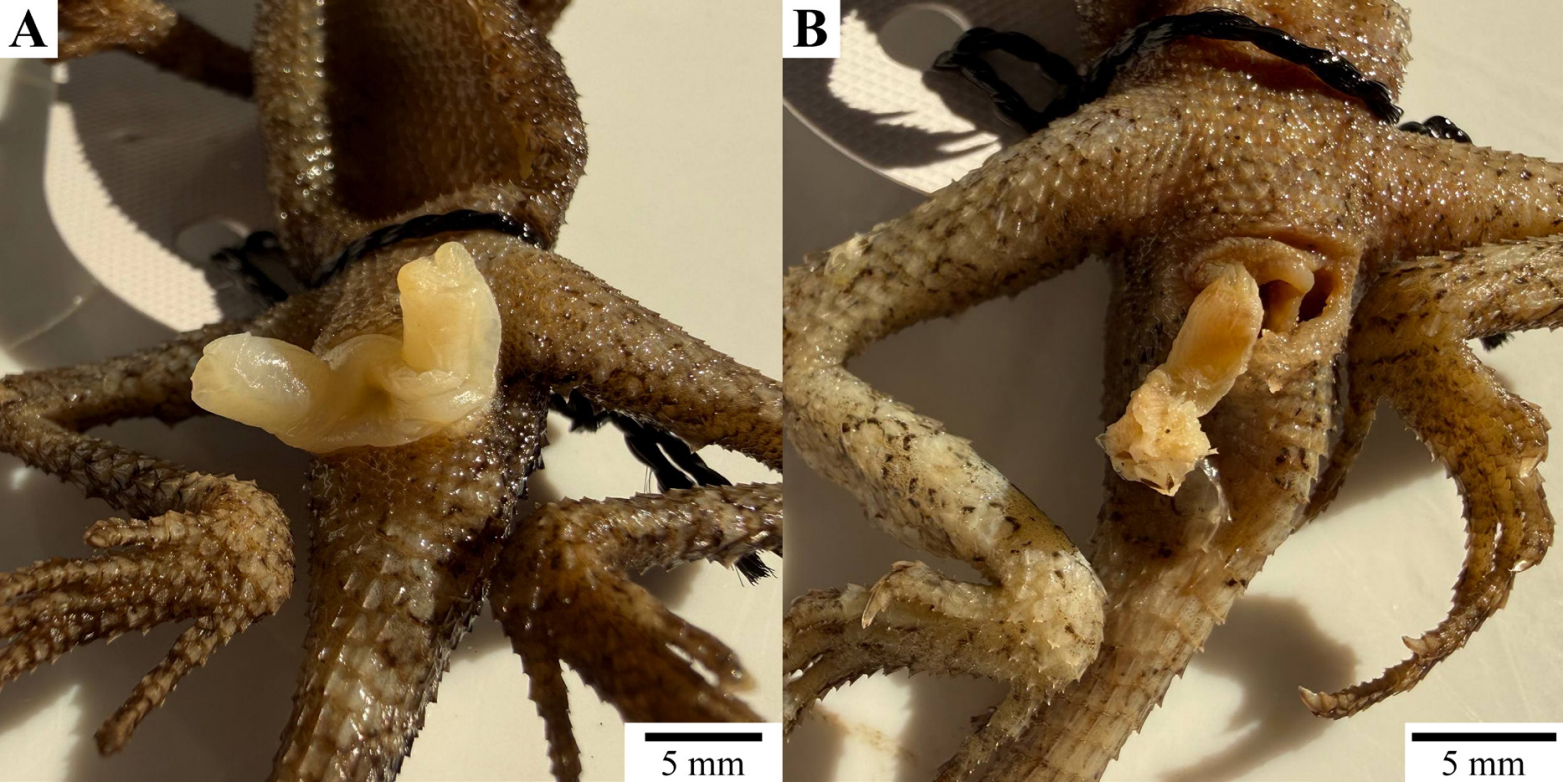
Cloaca opening with everted hemipenis of male *Acanthosaura syrax* sp. nov. **A**. Holotype THNHM31129; **B**. Paratype THNHM31135.

##### Diagnosis.

A small-sized species (maximum SVL 101.2 mm for females and 69.6 mm for males) with a single tiny conical spine above the posterior margin of the eye (maximum PS 2.7 mm for females and 2.0 mm for males); small spine on the occiput between the tympanum and the nuchal crest (maximum OS 4.1 mm for females and 2.4 mm for males); tympanum scaleless, small, roundish; less developed gular pouch; present small scales intermixed with three set of medium keeled scales line transversely from dorsal to lateral on the flanks; nuchal crest with six or seven tiny, keeled, compressed, asymmetrical triangular spines ascending from the first to the fifth or sixth, then decreasing in height; narrow diastema of 5–8 scales between the nuchal and vertebral crests; vertebral crest composed of small sized saw-like triangular scales beginning in the shoulder region and decreasing in size until the base of the tail; tail 1.06–1.16 of SVL (85.5–106.9 mm for females and 72.4–77.6 mm for males); black collar patch present; black eye patch absent.

##### Description of holotype.

Adult male. SVL 67.0 mm; TL 77.6 mm, tail complete; HL 21.1 mm; head length is one-third the length of the body (HL/SVL 0.32), narrow (HW/SVL 0.21), moderately tall (HD/HL 0.53), triangular in dorsal and lateral views; snout moderately short (SL/HL 0.33); rostrum wide (RW/RH 2.33), steeply sloping anteriorly; canthus rostralis prominent, forming a large projecting shelf extending above the eye, composed of 12 large scales; shelf terminates with a notch anterior to the postorbital spine; rostrum wide in size, rectangular, bordered laterally by the first supra labials (totally two scales from left and right) and posteriorly by four upper smaller scales; nasal roundish, surrounded by two prenasal anteriorly, three postnasals posteriorly and one subnasal; six scales between the nasal scales; elongate supra nasal scales; moderate scales above the orbit weakly keeled; three rows of slightly keeled scales below the orbit extending from the posterior margin of the nasal to post orbits; eye moderately large (EYE/HL 0.21), orbit moderately large (ORBIT/HL 0.35); prefrontal and frontal slightly keeled and smaller than the scales between the orbit and supralabials occipital scales weakly keeled; large parietal; tiny conical epidermal spine (1.30 mm) above the posterior margin of the eye, posteriorly pointed, surround by four small lanceolate scales; suborbital scales small, slightly keeled, extending in a row of five equal large scales from below the posterior margin of the eye to the anterior margin of the tympanum, decreasing in size posteriorly; tiny conical epidermal spine (1.80 mm) larger than the postorbital spine, posteriorly pointing backward, surrounded by a rosette of five small lanceolate scales; tympanum exposed, roundish similar to half of the eye (TD/EYE 0.56), surrounded by tiny rectangular scales; ten rectangular supralabials of similar size; mental pentagonal similar in size to the adjacent infralabials; two postmentals similar in size, four scales contacting the mental; chin shield large, extending posteriorly to the angle of the jaw, separated from the infralabials by one scales row anteriorly and four at the angle of the jaw; ten rectangular infralabials, scales slightly decreasing in size posteriorly; gular sharply keeled and spinose with a yellowish, small midventral row; dewlap and gular pouch very small; nuchal crest composed of seven tiny, keeled, compressed, asymmetrical triangular spines ascending from the first to the fifth or sixth, then decreasing in height, bordered on each side by two rows of keeled, triangular scales; nuchal crest followed by a diastema of eight scales at the base of the nape; dorsal body crest is half of the nuchal crest, extending from the posterior margin of the diastema onto the sacrum; vertebral crest composed of small, epidermal, flat, saw-like triangular scales, bordered by two rows of smaller paravertebral triangular scales; vertebral crest slightly decreasing to the sacrum, then fading progressively.

Body moderately developed, narrow, triangular in cross-section; dorsal body scales small intermixed with three set of medium keeled scales line transversely from dorsal to lateral on the flanks; scales of the pectoral region and abdomen larger than the dorsal scales, keeled, semi-transverse rows arranged; keeled scales anterior to the vent large; limbs moderately long, dorsal and ventral scales of forelimbs slightly keeled, proximal scales similar to the distal scales; five digits on the manus; subdigital scales keeled, 18 subdigital lamellae under the fourth finger. Scales on the hindlimb keeled, femoral scales slightly keeled and smaller than those on the tibia; five digits on the pes; subdigital scales keeled, 21 subdigital lamellae under the fourth toe; tail length 1.16 times SVL, tail covered with keeled spinose scales, keels on subcaudals directed posteriorly; subcaudals much longer than supracaudals; base of the tail 5.3 mm wide.

##### Variation.

The paratypes resemble the holotype in all aspects, except that females (THNHM31130 to 31134) differ from the holotype by lacking a transvers bar on the dorsal head region and possessing a pale yellowish-brown to orangish-brown body coloration. Females (THNHM31130–31134) exhibit sexual dimorphic in body size, being ~1.27 and ~1.23 times larger than males (holotype and THNHM31135 to 31136) in SVL and TAL, respectively. Morphometric and meristic data for the type series are presented in Table 2.

The hemipenis of three specimens (THNHM31129, 31135, 31136) were everted, revealing lengths of 5.3–7.2 mm. Each penis diverged into a symmetrical, sponged, budded-rosette shape with a tip width of 3.5–4.3 mm. In preserved ethanol, the hemipenis exhibited a creamy yellow coloration (Fig. 5).

Coloration in life is characterized by a dark brown head with creamy brown transverse bars on head with the most prominent crossing the orbital region. The lips and neck region are yellowish-green for males, and whitish-brown for females. Both sexes have no black eye patch. The male body presents a dark brown marbled reticulum interspersed with some pale greenish spots, whereas the female body display large, randomly distributed whitish-yellow patterns. Whitish or whitish-yellow ocellated spots are presented at the knee and elbow, with additional marking visible on the arm and leg. The ventral coloration is creamy. Arms exhibited darker and paler marks dorsally. Legs are darker dorsally with brown bars ventrally. The male tail is banded with pale brown and a dirty yellowish-rusty coloration, while the female tail is yellowish-creamy (Figs 1, 2, 3, 4).

##### Molecular description.

Molecular comparison, based on 698 nucleotides of the ND2 gene, reveal a minimal intraspecific variation among the eight analyzed specimens of *A. syrax* sp. nov. with pairwise sequence differences ranging from 0–0.01% (*n* = 8, GenBank PX738371–PX738378) (Table 3). However, the analysis demonstrated a low level of interspecific divergence between *A. syrax* sp. nov. and its seven congeners from Thailand, with genetic distance ranging from 0.14 to 0.21%. The genetic distances separating *A. syrax* sp. nov. from other *Acanthosaura* species were substantial, ranging from: 0.16% from *A. armata* (*n* = 2, GenBank AB266452, NC014175); 0.17–0.18% from *A. aurantiacrista* (*n* = 7, GenBank MH777406, MK798128–MK798133); 0.17% from *A. capra* Günther, 1861 (*n* = 1, GenBank AF128498); 0.16–0.18% from *A. cardamomensis* (*n* = 2, GenBank GU817397, GU817400); 0.17–0.21% from *A. crucigera* (*n* = 5, GenBank GU817389, HM143889, MH777402, MH777403, MH777408); 0.14–0.16% *A. lepidogaster* (*n* = 2, GenBank AF128499, KR092427); 0.20% from *A. meridiona* (*n* = 3, GenBank MH777404, MH777405, MH777407).

**Table:** **Table 2**. Morphometrical (in mm) and meristic data for the type series of *Acanthosaura syrax* sp. nov. For character abbreviations see Materials and methods. Meristic characters are given in the left column. NA = not applicable.

**Characteristics**	**Holotype THNHM31129**	**Paratype THNHM31130**	**Paratype THNHM31131**	**Paratype THNHM31132**	**Paratype THNHM31133**	**Paratype THNHM31134**	**Paratype THNHM31135**	**Paratype THNHM31136**	**Ranges**	
Sex	Male	Female	Female	Female	Female	Female	Male	Male	Male	Female
SVL	67.0	101.2	89.20	76.2	80.9	75.5	62.5	69.6	62.5–67.0	75.5–101.2
TaL	77.6	106.9	96.40	87.1	86.4	85.5	72.4	76.1	72.4–77.6	85.5–106.9
Tal/SVL	1.16	1.06	1.08	1.14	1.07	1.13	1.16	1.09	1.09–1.16	1.06–1.14
TBW	5.3	8.1	10.3	5.4	7.2	8.0	6.6	5.8	5.3–6.6	5.4–10.3
HL	21.1	30.4	29.7	23.5	24.4	24.7	20.5	20.3	20.3–21.1	23.5–30.4
HL/SVL	0.31	0.30	0.33	0.31	0.30	0.33	0.33	0.29	0.29–0.33	0.30–0.33
HW	14.1	21.2	21.4	17.5	16.7	17.7	15.6	14.4	14.1–15.6	16.7–21.4
HW/SVL	0.21	0.21	0.24	0.23	0.21	0.23	0.25	0.21	0.21–0.25	0.21–0.24
HW/HL	0.67	0.70	0.72	0.74	0.68	0.72	0.76	0.71	0.67–0.76	0.68–0.74
HD	11.1	17.6	16.8	12.7	14.4	14.9	11.8	10.7	10.7–11.8	12.7–17.6
HD/SVL	0.17	0.17	0.19	0.17	0.18	0.20	0.19	0.15	0.15–0.19	0.17–0.20
SL	7.0	8.0	9.9	7.8	7.8	7.9	6.7	6.7	6.7–7.0	7.8–9.9
SL/SVL	0.10	0.08	0.11	0.10	0.10	0.10	0.11	0.10	0.10–0.11	0.08–0.11
SL/HL	0.29	0.29	0.29	0.29	0.29	0.29	0.29	0.29	0.29	0.29
ORBIT	7.3	7.6	8.0	8.4	8.4	8.2	7.0	7.7	7.0–7.7	7.6–8.4
ORB/HL	0.11	0.08	0.09	0.11	0.10	0.11	0.11	0.11	0.11	0.08–0.11
EYE	4.5	5.2	5.2	5.2	5.1	5.3	5.2	5.0	4.5–5.2	5.1–5.3
EYE/HL	0.21	0.17	0.18	0.22	0.21	0.21	0.25	0.25	0.21–0.25	0.17–0.22
TD	2.5	3.2	3.1	2.5	2.2	2.7	1.9	2.1	1.9–2.5	2.2–3.2
TD/HL	0.16	0.16	0.16	0.16	0.16	0.16	0.16	0.16	0.16	0.16
TD/HD	0.23	0.18	0.18	0.20	0.15	0.18	0.16	0.20	0.16–0.23	0.15–0.20
TN	0	0	0	0	0	0	0	0	0	0
PS	1.3	2.3	2.7	1.9	1.6	2.2	1.8	2.0	1.3–2.0	1.6–2.7
PS/HL	0.06	0.08	0.09	0.08	0.07	0.09	0.09	0.10	0.06–0.10	0.07–0.09
NSL	2.4	4.7	4.1	3.0	3.2	3.5	2.4	2.4	2.4	3.0–4.7
NSL/HL	0.11	0.15	0.14	0.13	0.13	0.14	0.12	0.12	0.11–0.12	0.13–0.15
NN	7	7	7	7	7	7	7	7	7	7
DS	1.5	2.6	1.4	1.4	1.4	1.6	1.4	1.0	1.0–1.5	1.4–2.6
DS/HL	0.07	0.09	0.05	0.06	0.06	0.06	0.07	0.05	0.05–0.07	0.05–0.09
NSW	0.6	1.1	1.3	0.5	1.0	0.7	0.4	0.5	0.4–0.6	0.5–1.3
NSW/NSL	0.25	0.23	0.32	0.17	0.31	0.20	0.17	0.21	0.17–0.25	0.17–0.32
DIAS	3.8	6.7	4.40	3.3	4.2	3.1	3.3	4.1	3.3–4.1	3.1–6.7
DIAS/SVL	0.06	0.07	0.05	0.04	0.05	0.04	0.05	0.06	0.05–0.06	0.04–0.07
DIASN	8	8	8	7	5	6	7	7	7–8	5–8
FOREL	29.1	44.5	39.50	35.3	34.4	31.7	27.9	33.1	27.9–33.1	31.7–44.5
FOREL/SVL	0.43	0.44	0.44	0.46	0.43	0.42	0.45	0.48	0.43–0.48	0.42–0.46
HINDL	33.4	49.3	45.40	39.8	38.8	37.3	33.8	35.9	33.4–35.9	37.3–49.3
HINDL/SVL	0.50	0.49	0.51	0.52	0.48	0.49	0.54	0.52	0.50–0.54	0.48–0.52
FOREL/HINDL	0.87	0.90	0.87	0.89	0.89	0.85	0.83	0.92	0.83–0.92	0.85–0.90
SUPRAL	10	11	10	9	10	10	11	10	10–11	9–11
INFRAL	10	11	10	10	11	10	10	10	10	10–11
VENT	60	58	57	60	59	56	56	58	56–60	56–60
FI	18	19	19	20	17	17	17	18	17–18	17–20
TO	21	19	20	19	19	17	21	22	21–22	17–20
OS	1.8	4.1	2.60	2.9	2.6	2.6	2.4	1.9	1.8–2.4	2.6–4.1
PS/OS	0.72	0.56	1.04	0.66	0.62	0.85	0.75	1.05	0.72–1.05	0.56–1.04
OS/HL	0.09	0.13	0.09	0.12	0.11	0.11	0.12	0.09	0.09–0.12	0.09–0.13
NSSOS	5	6	5	5	6	6	5	5	5	5–6
CS	12	13	14	12	13	13	12	12	12	12–14
RW	2.1	3.5	2.9	2.0	2.1	3.3	1.8	1.3	1.3–2.1	2.0–3.5
RH	0.9	1.5	1.1	1.3	0.8	1.2	0.7	0.8	0.7–0.9	0.8–1.5
RS	6	7	7	6	6	7	6	6	6	6–7
NS	6	7	5	6	6	7	6	6	6	5–7
NCS	8	7	8	8	9	8	8	8	8	7–9
NSCSL	7	7	7	7	8	6	7	6	6–7	6–8
NR	2	2	2	2	2	2	2	2	2	2
NSSLC	10	10	10	9	9	10	9	9	9–10	9–10
MW	1.1	1.7	1.0	1.8	1.0	1.2	0.7	1.0	0.7–1.1	1.0–1.8
MH	0.7	1.5	1.2	1.4	1.2	1.2	0.7	1.1	0.7–1.1	1.2–1.5
MW/MH	1.57	1.13	0.83	1.29	0.83	1.00	1.00	0.91	0.91–1.57	0.83–1.29
PM	4	4	4	4	4	4	4	4	4	4
YAS	1	1	1	1	1	1	1	1	1	1
ND	1	1	1	1	1	1	1	1	1	1
LKP	1	1	1	1	1	1	1	1	1	1
BEP	0	0	0	0	0	0	0	0	0	0
ESBO	0	0	0	0	0	0	0	0	0	0
GP	1	0	1	0	0	0	0	0	0	0
OF	1	1	1	1	1	1	1	1	1	1
NEBO	9	10	9	10	10	9	9	10	9–10	9–10
NSSOB	4	4	5	5	4	4	4	4	4	4–5
PL	5.3	NA	NA	NA	NA	NA	7.0	7.2	5.3–7.2	NA
PW	3.8	NA	NA	NA	NA	NA	3.5	4.3	3.5–4.3	NA
EGG	NA	6	NA	16	11	6	NA	NA	NA	6–16

Furthermore, the combined Maximum–Likelihood (ML) and Bayesian Inference (BI) phylogenetic analyses of the ND2 gene strongly supported the distinct taxonomic status of *A. syrax* sp. nov. (76/0.78). The reconstructed phylogenies were topologically consistent, recovering the new species as a highly supported monophyletic clade completely separated from all other included taxa. Importantly, the robust nodal support specifically confirms the evolutionary divergence between *A. syrax* sp. nov. and its morphologically similar congener, *A. lepidogaster* (Fig. 7).

**Figure 6.**
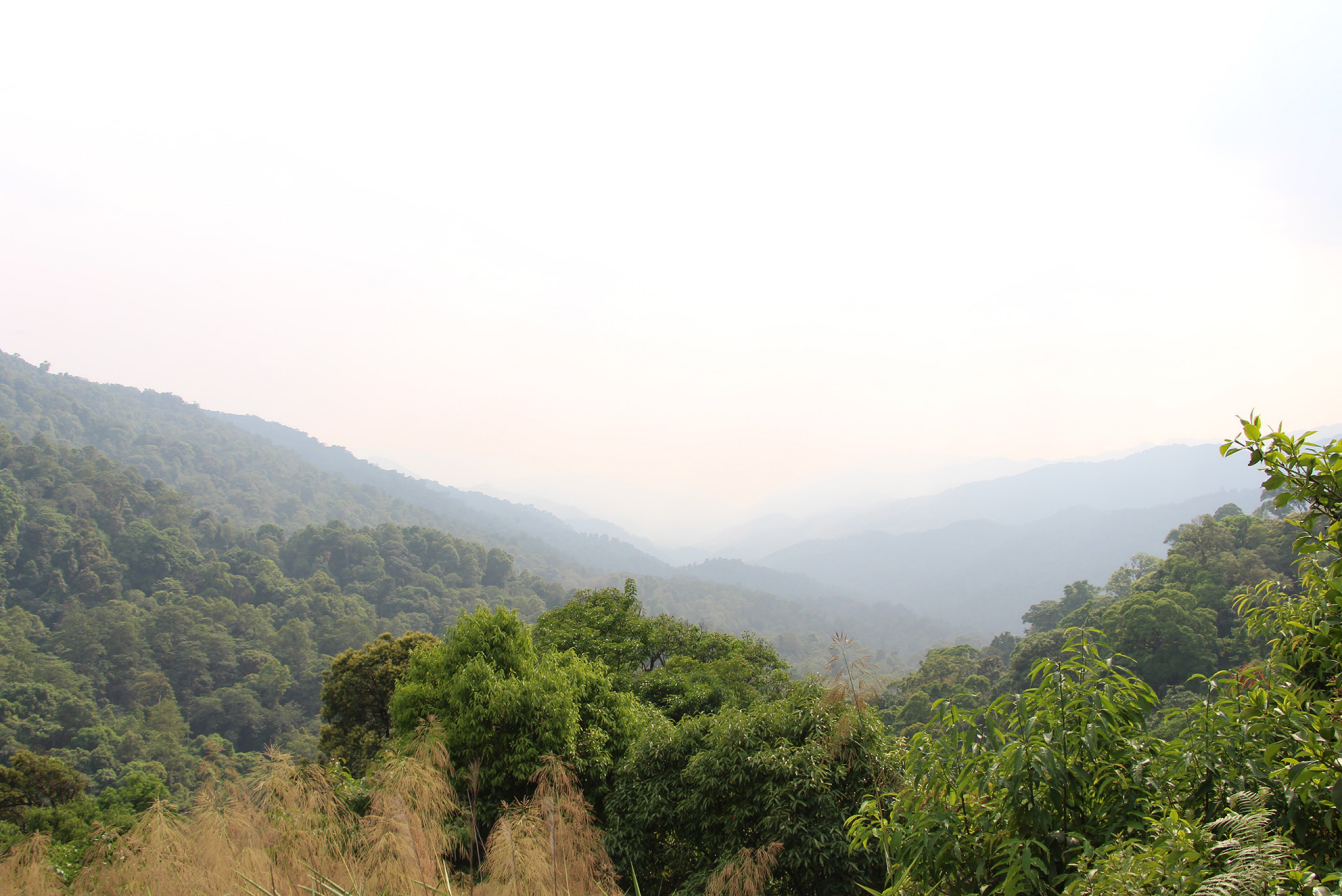
Forest habitat of *Acanthosaura syrax* sp. nov. in Mae Wong National Park, Mae Wong District, Kampheang Phet Province, Thailand (16°05'43.5"N, 99°07'25.2"E), at 1,340 m a.s.l.

**Figure 7.**
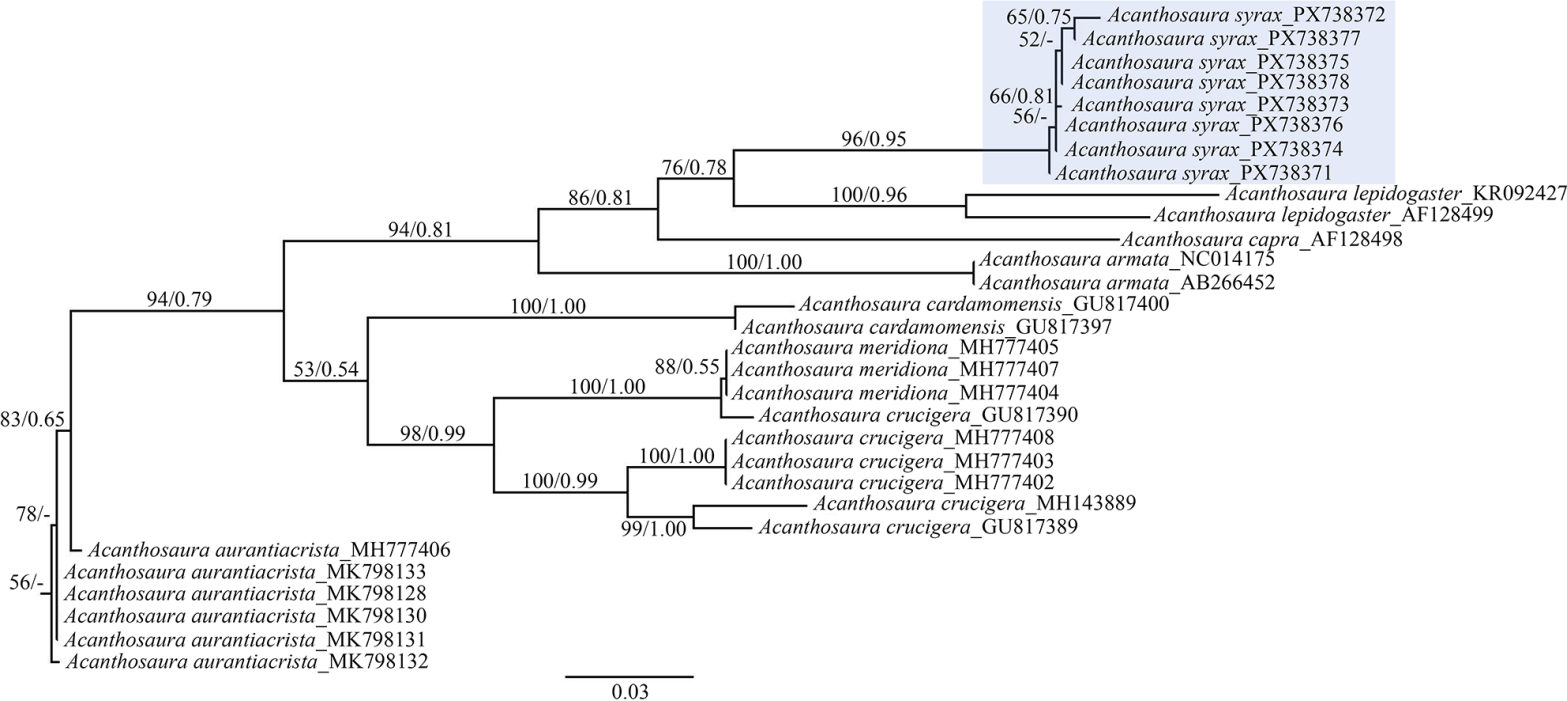
Phylogram of investigated members of *Acanthosaura* inferred from the ND2 gene. Nodal numbers represent Maximum Likelihood bootstrap support values (BS, left) and Bayesian Inference posterior probabilities (PP, right). Values below 50% for ML or 0.70 for BI are indicated by a hyphen (-) or are not shown.

Additionally, molecular comparison, based on 600 nucleotides of the COI gene, also reveal minimal intraspecific variation among the eight analyzed specimens of *A. syrax* sp. nov. with pairwise sequence differences ranging from 0–0.34% (*n* = 8, GenBank PZ164171–PZ164178) (Table 4). In contrast, the analysis demonstrated high levels of interspecific divergence between *A. syrax* sp. nov. and its 14 congeners (mostly from Vietnam), with genetic distances ranging from 14.96% to 28.13%. The genetic distances separating *A. syrax* sp. nov. from other *Acanthosaura* species were substantial, ranging from: 17.30–17.75% from *A. brachypoda* Ananjeva, Orlov, Nguyen & Ryabov, 2011 (*n* = 4, GenBank MK695182–MK695185); 14.96–15.44% from *A. capra* (*n* = 1, GenBank MK239022); 16.82–17.25% from *A. crucigera* (*n* = 3, GenBank MT607980–MT607982); 25.68–27.14% from *A. murphyi* Nguyen, Do, Hoang, Nguyen, McCormack, Nguyen, Orlov, Nguyen & Nguyen, 2018 (*n* = 3, GenBank MK239025–MK239027); 25.82–26.58% from *A. coronata* Günther, 1861 (*n* = 5, GenBank MK695186–MK695190); 26.74–27.45% from *A. cuongi* Ngo, Le, Nguyen, Nguyen, Nguyễn, Phan, Nguyen, Ziegler & Do, 2025 (*n* = 3, GenBank PP669985–PP669987); 27.64–28.13% from *A. grismeri* Le, Nguyen, Nguyen, Ziegler, Do & Ngo, 2025 (*n* = 3, GenBank PV646694–PV646696); 16.64–17.53% from *A. lepidogaster* (*n* = 5, GenBank MK695191–MK695195); 19.08–19.55% from *A. liui* Liu, Hou, Mo & Rao, 2020 (*n* = 5, GenBank MT769763–MT769767); 17.90–18.34% from *A. longicaudata* Liu, Rao, Hou, Orlov, Ananjeva & Zhang, 2022 (*n* = 4, GenBank ON207528–ON207531); 16.18–17.04% from *A. rubrilabris* (*n* = 6, GenBank ON207534–ON207537, ON207543–ON207544); 20.04–20.74% from *A. nataliae* Orlov, Nguyen & Nguyen, 2006 (*n* = 3, GenBank MK695202–MK695204); and 15.75–16.19% from *A. phongdienensis* Nguyen, Jin, Dinh, Nguyen, Zhou, Che, Murphy & Zhang, 2019 (*n* = 6, GenBank MK695205–MK695210).

**Table 3.** Percentage pairwise genetic distances based on the ND2 gene of among and within nine species of the genus *Acanthosaura*, including the outgroup *Calotes emma* (see Appendix 1: Table A1 for more details of samples used in the study).

**Taxa**	**1**	**2**	**3**	**4**	**5**	**6**	**7**	**8**	**9**
*Calotes emma*	–								
*Acanthosaura syrax* sp. nov.	1.91–1.94	0.00–0.01							
*Acanthosaura armata*	2.17	0.16	0.00						
*Acanthosaura aurantiacrista*	2.22–2.25	0.17–0.18	0.16	0.00					
*Acanthosaura capra*	2.19	0.17	0.18	0.19–0.20	–				
*Acanthosaura cardamomensis*	2.21–2.29	0.16–0.18	0.17–0.18	0.13–0.15	0.20–0.22	0.01			
*Acanthosaura crucigera*	1.96–2.17	0.17–0.21	0.16–0.22	0.01–0.15	0.19–0.24	0.13–0.16	0.00–0.14		
*Acanthosaura lepidogaster*	1.96–2.12	0.14–0.16	0.18–0.20	0.18–0.20	0.17–0.19	0.18–0.23	0.18–0.25	0.09	
*Acanthosaura meridiona*	2.09	0.20	0.20	0.12	0.22	0.15–0.16	0.01–0.11	0.21–0.24	0.00

**Table 4.** Percentage pairwise genetic distances based on the COI gene of among and within nine species of the genus *Acanthosaura*, including the outgroup *Calotes emma* (see Appendix 1: Table A1 for more details of samples used in this study).

**Taxa**	**1**	**2**	**3**	**4**	**5**	**6**	**7**	**8**	**9**	**10**	**11**	**12**	**13**	**14**	**15**
*Calotes versicolor*	–														
*Acanthosaura syrax* sp. nov.	26.83–27.09	0.00–0.34													
*Acanthosaura brachypoda*	28.63–29.16	17.30–17.75	0.00–0.54												
*Acanthosaura capra*	27.34	14.96–15.44	25.08–25.32	0.00											
*Acanthosaura crucigera*	29.68	16.82–17.25	17.98–18.20	25.68	0.00										
*Acanthosaura murphyi*	27.34–28.37	25.68–27.14	25.57–26.58	6.62–6.81	24.72–25.20	0.17–1.03									
*Acanthosaura coronata*	27.60–28.63	25.82–26.58	27.09–28.11	18.43–19.58	25.32–25.82	19.12–20.27	0.00–2.35								
*Acanthosaura cuongi*	30.74	26.74–27.45	27.09–27.60	17.37–17.72	26.00–26.46	18.25–18.59	15.75–16.42	0.00–0.35							
*Acanthosaura grismeri*	29.07–29.16	27.64–28.13	29.07–29.42	19.30–19.44	27.76–27.95	20.56–21.46	8.25–8.56	16.42–16.85	0.00						
*Acanthosaura lepidogaster*	29.68–30.74	16.64–17.53	4.01–4.77	24.58–25.57	17.08–17.98	24.09–25.82	25.57–27.09	26.58–27.34	26.73–27.09	0.00–1.62					
*Acanthosaura liui*	30.41–30.68	19.08–19.55	21.94–22.43	26.70–26.96	18.38–18.62	26.44–27.22	28.53–29.87	25.93–26.44	28.80–29.06	19.55–20.02	0.00–0.18				
*Acanthosaura longicaudata*	29.16	17.90–18.34	10.26	23.54	18.12	23.54–24.01	24.83–25.57	26.71–26.95	24.95–25.68	9.45–10.26	21.22–21.46	0.00			
*Acanthosaura rubrilabris*	30.21–30.48	16.18–17.04	13.38–14.02	24.72–25.20	16.82–17.47	24.49–25.92	24.58–25.82	23.83–24.77	25.68–26.98	12.33–13.59	18.38–19.32	13.67–14.09	0.00–0.86		
*Acanthosaura nataliae*	34.87–35.15	20.04–20.74	20.04–20.50	31.28–31.55	19.81–20.04	30.74–31.28	29.95–30.74	30.74–31.28	30.74–30.95	20.74–21.21	23.41–23.91	20.50–20.74	19.81–20.97	0.00–0.18	
*Acanthosaura phongdienensis*	29.95–30.74	15.75–16.19	14.88–15.10	25.57–26.08	18.20–18.66	25.08–26.08	25.08–26.08	27.09–27.60	27.60–28.28	13.81–14.88	19.79–20.50	12.96	5.15–6.10	22.16–22.40	0.00–0.54

**Figure 8.**
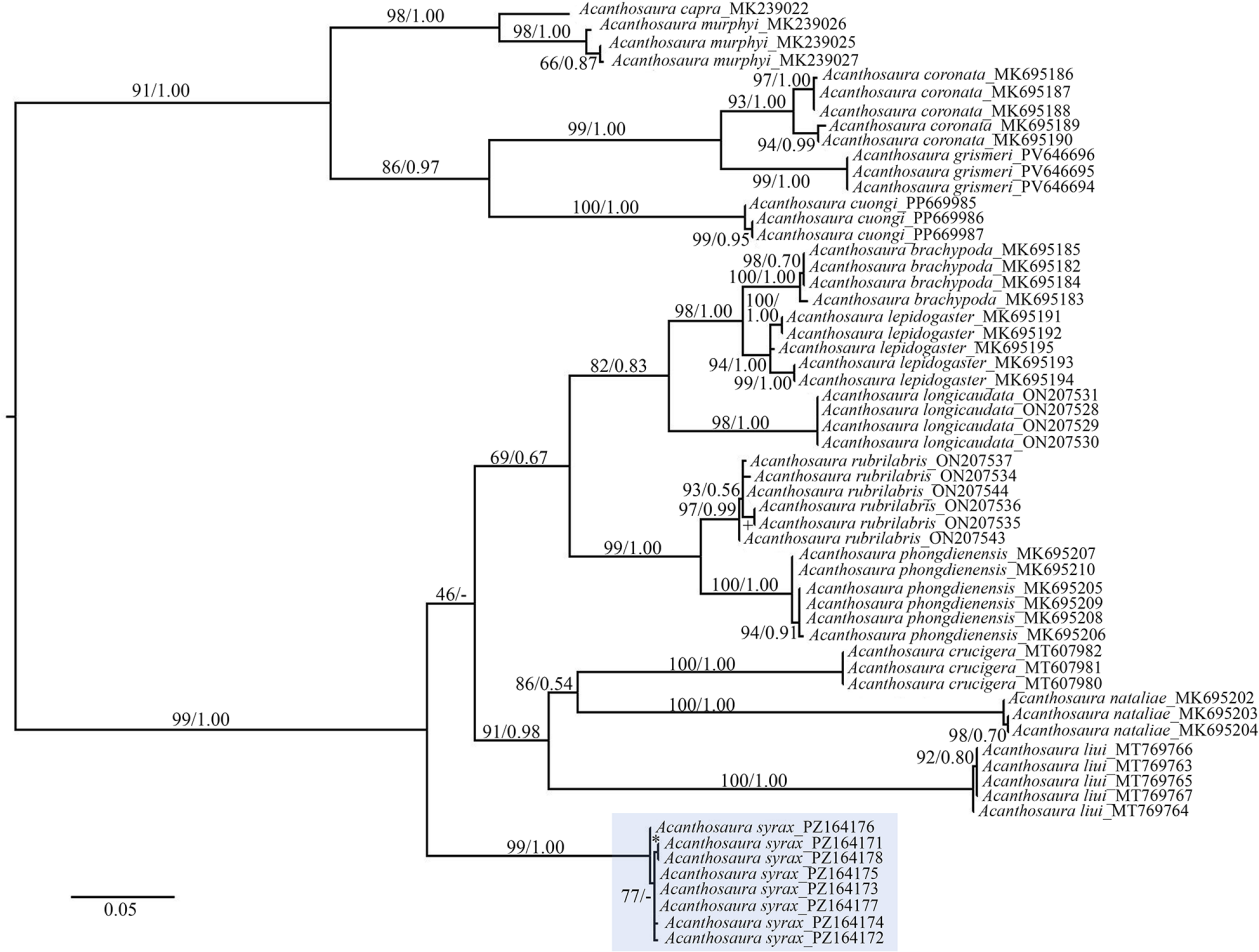
Phylogram of investigated members of *Acanthosaura* inferred from the COI gene. Nodal numbers represent Maximum Likelihood bootstrap support values (BS, left) and Bayesian Inference posterior probabilities (PP, right). Values below 50% for ML or 0.70 for BI are indicated by a hyphen (-) or are not shown. * = 96/0.83, + = 99/0.99.

These significant divergence values firmly support the species distinct taxonomic status. Concordant with the ND2 results, the dual ML and BI phylogenetic analyses of the COI dataset recovered *A. syrax* sp. nov. as an independent, well-supported monophyletic lineage (99/1.00) that is clearly distinguished from all other sampled *Acanthosaura* taxa (Fig. 8).

##### Natural history.

This species has a restricted altitudinal distribution within the Mae Wong National Park, where it is typically observed on the upper mountain range, exceeding 1,300 m a.s.l. (Fig. 6). Based on field observations, our team did not encounter any individuals below 1,000 m a.s.l. The lizard adheres to a clear diurnal activity pattern, dedicating the early to mid-day hours to basking behavior on exposed substrates such as rocks, fallen logs, small shrubs, or cement structures. Once the sun begins to set in the afternoon, the individual retreats into the forest to secure nocturnal rest sites under logs, rocks, or within tree cavities. Behaviorally, the species exhibits a low activity during the day relative immobility allowed authors to approach and capture individuals manually without inducing an escape response. Some individuals engage in opportunistic foraging, with observations of some individuals consuming earthworms on the ground during the dawn period. Additionally, the species displays marked seasonality in its activity levels, being most frequently encountered and exhibiting high intensity during the late dry season and the transition to the early monsoon (April to mid-June). Following this peak, activity levels decline as the monsoon progresses and temperatures drop, becoming significantly lower during the cold season (November–December) when minimum temperatures fall to approximately 10 °C. During the rainy season, sightings were primarily restricted to midday periods under direct sunlight, suggesting that solar radiation becomes a limiting factor for activity during the wetter, cooler months.

##### Distribution.

*Acanthosaura syrax* sp. nov. occurs in northwestern Thailand according to personal field observations restricted to areas exceeding 1,000 m a.s.l. in Mae Wong National Park, Tak–Kamphaeng Phet Province, Thailand (16°06'01.6"N, 99°06'28.0"E).

##### Etymology.

The specific epithet syrax is a noun in apposition, named in reference to the dragon “Syrax”, the mount of Queen Rhaenyra Targaryen. This name is inspired by the species’ distinctive yellow-hued coloration, its relatively diminutive size compared to its congeners, and its significant reproductive output, reflected in its high clutch size. We suggest the following common names, “Syrax mountain horned dragon” (English) and “King Ka Khaow Naam Lek Syrax” (กิ้งก่าเขาหนามเล็กไซแรกซ์) (Thai).

##### Comparisons.

Suppl. material 1: table SS1 summarizes the comparisons of the morphometric measurements and meristic data for all currently recognized species in comparison with *A. syrax* sp. nov. and other recognized *Acanthosaura* species. Morphological comparison data of *Acanthosaura* species in Thailand are compared with *A. armata*, *A. aurantiacrista*, *A. cardamomensis*, *A. crucigera*, *A. lepidogaster*, *A. meridiona*, and *A. phuketensis* based on published data by Wood Jr et al. (2010), Pauwels et al. (2015), Trivalairat et al. (2020, 2022). Although *A. rubrilabris* is distributed in the northern region of Thailand, the morphological evidence provided by from Liu et al. (2022) is based exclusively on specimens from China. Consequently, we excluded this species from our comparisons with other Thai congeners.

*Acanthosaura syrax* sp. nov. differs from *A. armata* in presenting a smaller TaL/SVL ratio (1.06–1.16 vs 1.20–1.60), smaller PS/HL ratio (0.06–0.10 vs 0.22–0.58), smaller NSL/HL ratio (0.11–0.15 vs 0.22–0.51), smaller DS/HL ratio (0.05–0.09 vs 0.20–0.52), smaller OS/HL ratio (0.09–.13 vs 0.16–0.43), fewer NS (7 vs 12), fewer INFRAL (10–11 vs 12–15), and fewer NCS (7–9 vs 10–17).

*Acanthosaura syrax* sp. nov. differs from *A. aurantiacrista* in presenting a smaller TaL/SVL ratio (1.06–1.16 vs 1.40–1.70), smaller SL/HL (0.29 vs 0.41–.58), smaller PS/HL ratio (0.06–0.10 vs 0.24–0.84), smaller NSL/HL ratio (0.11–0.15 vs 0.35–0.95), smaller DS/HL ratio (0.05–0.09 vs 0.15–0.38), greater NSW/NSL (0.17–.32 vs 0.07–0.14), smaller OS/HL ratio (0.09–.13 vs 0.19–0.44), greater FOREL/HINDL (0.83–0.92 vs 0.75–0.80), fewer VENT (56–60 vs 63–66), fewer TO (17–22 vs 25–29), smaller PS/OS (0.56–1.05–1.08–1.91), fewer NS (7 vs 8), and fewer NCS (7–9 vs 11–13).

*Acanthosaura syrax* sp. nov. differs from *A. cardamomensis* in presenting a smaller SL/HL (0.29 vs 0.47–0.57), smaller PS/HL ratio (0.06–0.10 vs 0.14–0.45), smaller NSL/HL ratio (0.11–0.15 vs 0.17–0.66), smaller DS/HL ratio (0.05–0.09 vs 0.14–0.45), smaller NSW (0.4–1.3 vs 1.8–4.2), smaller OS/HL ratio (0.09–0.13 vs 0.24–0.56), slightly fewer NS (5–7 vs 7–10), slightly fewer NCS (7–9 vs 9–17), slightly fewer NSSLC (9–10 vs 10–19), and the absence of black eye patch.

*Acanthosaura syrax* sp. nov. differs from *A. crucigera* in presenting of a smaller ORBIT (7.0–8.4 vs 8.9–10.8), slightly smaller PS/HL ratio (0.06–0.10 vs 0.09–0.33), slightly smaller DS/HL ratio (0.05–0.09 vs 0.09–0.24), slightly smaller NSW (0.4–1.3 vs 1.3–3.4), fewer DIASN (5–8 vs 9–25), slightly fewer NCS (7–9 vs 9–12), slightly fewer NSSLC (9–10 vs 10–14), and the absence of black eye patch.

*Acanthosaura syrax* sp. nov. is most closely related to *A. lepidogaster*. *Acanthosaura syrax* sp. nov. differs from *A. lepidogaster* in presenting a smaller TaL/SVL ratio (1.06–1.16 vs 1.6–1.9), greater HD (14.9–17.6 vs 12.0–12.5), fewer DIASN (5–8 vs 10–14), slightly fewer TO (17–22 vs 22–23), greater OS/HL ratio (0.09–0.13 vs 0.14–0.15), and fewer PM (4 vs 5).

*Acanthosaura syrax* sp. nov. differs from *A. meridiona* in presenting a smaller TaL (72.4–106.9 vs 108.5–176.0), greater HL (24.7–30.4 vs 17.0–24.1), smaller ORBIT/HL ratio (0.08–0.11 vs 0.44–0.53), smaller PS/HL ratio (0.06–0.10 vs 0.16–0.38), slightly smaller NSL/HL ratio (0.11–0.15 vs 0.15–0.37), fewer DIASN (5–8 vs 10–16), slightly fewer VENT (56–60 vs 60–68), slightly smaller OS/HL ratio (0.09–0.13 vs 0.13–0.25), fewer NCS (7–9 vs 10–15), fewer NSCSL (6–8 vs 9–11), and more NR (2 vs 1).

*Acanthosaura syrax* sp. nov. differs from *A. phuketensis* in presenting a smaller TaL/SVL ratio (1.06–1.16 vs 1.40–1.70), smaller TD (1.9–3.2 vs 3.5–4.7), smaller PS/HL (0.06–0.10 vs 0.23–0.38), smaller NSL/HL ratio (0.11–0.15 vs 0.21–0.39), smaller DS/HL ratio (0.05–0.09 vs 0.11–0.26), smaller NSW (0.4–1.3 vs 1.4–2.9), fewer DIASN (5–8 vs 12–17), fewer NCS (7–9 vs 12–13), fewer NSSLC (9–10 vs 11–14), and the absence of black eye patch.

**Figure 9.**
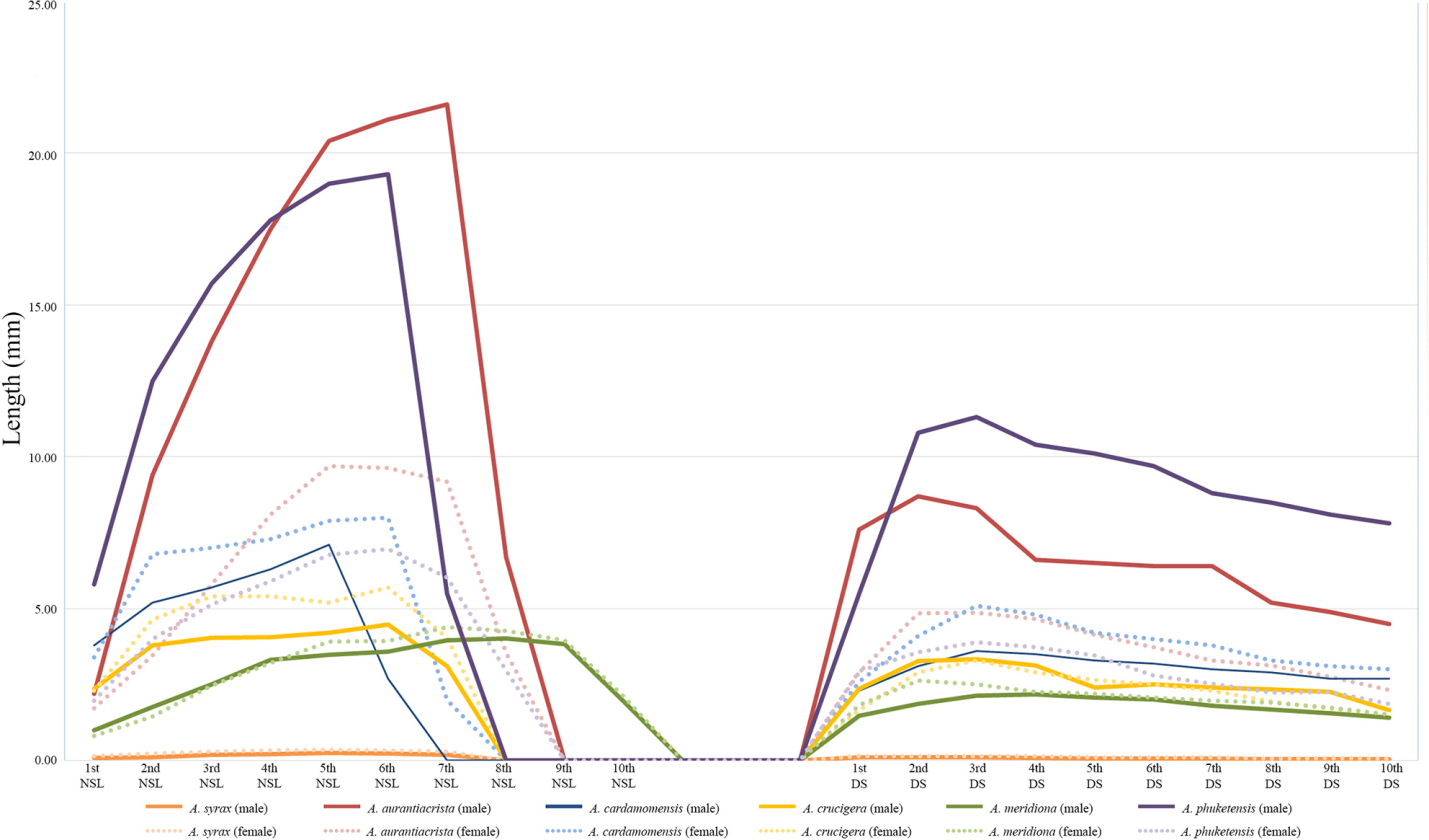
Average length of nuchal (NSL) and dorsal (DS) crests for six *Acanthosaura* species occurring in Thailand.

The results indicate that *A. crucigera* and *A. lepidogaster* mostly share characteristics with *A. syrax* sp. nov. However, a close examination led us to separate *A. syrax* sp. nov. from these similar species based on coloration, morphological characteristics, especially nuchal–dorsal crest patterns, as well as on molecular data (Fig. 9) (Suppl. material 1: table S2).

For other congeners outside Thailand, they are compared based on published data by Günther (1861), Orlov et al. (2006), Wood Jr et al. (2009), Ananjeva et al. (2011, 2020), Nguyen et al. (2018, 2019), Liu and Rao (2019), Liu et al. (2020, 2022), Le et al. (2025), and Ngo et al. (2025).

*Acanthosaura syrax* sp. nov. differs from *A. bintangensis* Wood, Grismer, Grismer, Northayati, Chan & Bauer, 2009 in presenting a smaller TaL/SVL ratio (1.06–1.16 vs 1.30–1.40), smaller NSL/HL ratio (0.11–0.15 vs 0.17–0.21), smaller NSW (0.4–1.3 vs 1.6–2.1), fewer DIASN (5–8 vs 11–15), fewer SUPRAL (9–11 vs 12), more VENT (56–60 vs 51–55), fewer FI (17–20 vs 23), fewer TO (17–22 vs 26–28), smaller RW (1.3–3.5 vs 3.6–5.3), smaller RH (0.7–1.5 vs 1.7–20), fewer NS (5–7 vs 8), fewer NCS (7–9 vs 10–11), more NR (2 vs 1), smaller GP (0–1 vs 3–4), the presence of LKP which is absent in from *A. bintangensis*, and the absence of black eye patch and elliptical scales below the orbit.

*Acanthosaura syrax* sp. nov. differs from *A. brachypoda* in presenting a smaller TaL/SVL ratio (1.30–1.40 vs 1.58), smaller SL (6.7–9.9 vs 12.2), smaller TD (1.9–3.2 vs 3.6), smaller PS/HL ratio (0.06–0.10 vs 0.11), smaller NSL/HL ratio (0.11–0.15 vs 0.16), smaller NSW (0.4–1.3 vs 1.6), fewer SUPRAL (9–11 vs 12–13), fewer VENT (56–60 vs 63), fewer TO (17–22 vs 24), greater OS/HL ratio (0.09–0.13 vs 0.03), smaller RH (0.7–1.5 vs 2.3), fewer NS (5–7 vs 9), smaller MW (0.7–1.8 vs 2.9), smaller MH (0.7–1.5 vs 2.1), and the absence of black eye patch.

*Acanthosaura syrax* sp. nov. differs from *A. capra* in presenting a smaller TaL/SVL ratio (1.06–1.16 vs 1.20–1.50), smaller TD (1.9–3.2 vs 3.4–5.2), smaller PS/HL ratio (0.06–0.10 vs 0.36), smaller NSL/HL ratio (0.11–0.15 vs 0.42–0.43), smaller DS/HL ratio (0.05–0.09 vs 0.16–0.17), smaller FOREL (27.9–44.5 vs 54.2–83.8), smaller HINDL (33.4–49.3 vs 78.5–107.2), fewer INFRAL (10–11 vs 12–13), smaller RW (1.3–3.5 vs 4.2–4.6), smaller RH (0.7–1.5 vs 1.8–2.3), fewer NS (5–7 vs 9), smaller MW (0.7–1.8 vs 1.9–2.2), smaller GP (0–1 vs 3–4), the presence of OS, NSSOS which is absent in *A. capra*, and the absence of black eye patch.

*Acanthosaura syrax* sp. nov. differs from *A. coronata* in presenting a greater TaL/SVL ratio (1.06–1.16 vs 0.6–1.0), greater HL (24.7–30.4 vs 14.4–16.3), greater HW (17.7–21.2 vs 13.6–17.5), fewer SUPRAL (9–11 vs 12–13), more FI (17–20 vs 13–14), greater RW (1.3–3.5 vs 0.8–0.9), fewer RS (6–7 vs 9), fewer NR (2 vs 3–4), and the presence of postorbital spines, nuchal scales, dorsal scales, diastema, occipital spines, and black, diamond shaped, nuchal collar, which is absent in *A. coronata*.

*Acanthosaura syrax* sp. nov. differs from *A. cuongi* in presenting a smaller TaL (72.4–106.9 vs 113.9–132.6), slightly greater HW/SVL (0.21–0.25 vs 0.18–0.21), smaller ORBIT/HL (0.08–0.11 vs 0.27–0.38), smaller EYE (4.5–5.3 vs 6.2–7.5), slightly greater PS/HL (0.06–0.10 vs 0.00–0.06), greater NSL/HL (0.11–0.15 vs 0.06–0.10), smaller NSW/NSL (0.17–0.32 vs 0.41–0.76), fewer NS (7 vs 8), greater FOREL/SVL (0.42–0.48 vs 0.31–0.38), greater FOREL/HINDL (0.83–0.92 vs 0.70–0.78), slightly more FI (17–20 vs 14–17), greater OS/HL (0.09–0.13 vs 0.04–0.08), greater CS (12–14 vs 10–11), fewer NS (5–7 vs 8–10), greater NR (2 vs 1), and the absence of black eye patch.

*Acanthosaura syrax* sp. nov. differs from *A. grismeri* in presenting a smaller TaL/SVL (72.4–106.9 vs 1.21–1.44), TBW (5.3–10.3 vs 11.1–13.0), SL/HL (0.29 vs 0.31–0.40), ORBIT/HL (0.08–0.11 vs 0.32–0.43), TD (1.9–3.2 vs 3.2–5.3), NSW/NSL (0.17–0.32 vs 0.60–1.04), OS/HL (0.09–0.13 vs 0.40–1.94), greater HW/SVL (0.21–0.25 vs 0.19–0.20), DS/HL (0.05–0.09 vs 0.03–0.04), FOREL/SVL (0.42–0.48 vs 0.29–0.34), FOREL/HINDL (0.83–0.92 vs 0.66–0.77), FI (17–20 vs 12–16), PS/OS (0.56–1.05 vs 0.04–0.09), RS (6–7 vs 4–5), the presence diastema and oblique fold.

*Acanthosaura syrax* sp. nov. differs from *A. liui* in presenting a smaller TaL/SVL ratio (1.06–1.16 vs 1.47–1.77), smaller TBW (5.3–10.3 vs 10.6–13.9), smaller SL (6.7–9.9 vs 10.3–11.3), slightly smaller ORBIT (7.0–8.4 vs 8.4–8.9), smaller EYE (4.5–5.3 vs 5.8–6.4), greater TD/HD ratio (0.15–0.23 vs 0.03–0.04), smaller NSL/HL ratio (0.11–0.15 vs 0.17–0.24), smaller DS/HL ratio (0.05–0.09 vs 0.10–0.12), smaller HINDL (33.4–49.3 vs 50.7–52.6), slightly fewer TO (17–22 vs 22–25), fewer RS (6–7 vs 8–9), smaller MH (0.7–1.5 vs 1.7–2.3), fewer PM (4 vs 5–6), smaller GP (0–1 vs 2–3), and the absence of black eye patch.

*Acanthosaura syrax* sp. nov. differs from *A. longicaudata* in presenting a smaller TaL/SVL ratio (1.06–1.16 vs 1.62–2.04), greater HW (17.7–21.2 vs 14.2–17.4), smaller SL/HL ratio (0.29 vs 0.35–0.37), fewer ORBIT/HL (0.08–0.11 vs 0.32–0.37), slightly greater TD (1.9–3.2 vs 1.4–1.9), smaller NSW/NSL ratio (0.17–0.32 vs 0.46–0.64), HINDL/SVL ratio (0.48–0.54 vs 0.55–0.63), FOREL/HINDL ratio (0.83–0.92 vs 0.64–0.75), and slightly more VENT (56–60 vs 47–56).

*Acanthosaura syrax* sp. nov. differs from *A. murphyi* in presenting a smaller TaL/SVL ratio (1.06–1.16 vs 1.48–1.54), smaller HD (14.9–17.6 vs 18.5–20.6), smaller SL (6.7–9.9 vs 10.3–15.3), smaller ORBIT (7.0–8.4 vs 9.9–12.3), smaller PS/HL ratio (0.06–0.10 vs 0.16–0.34), smaller NSL/HL ratio (0.11–0.15 vs 0.24–0.43), smaller DS/HL ratio (0.05–0.09 vs 0.14–0.51), smaller NSW (0.4–1.3 vs 2.9–4.8), smaller FOREL (27.9–44.5 vs 49.8–56.6), smaller HINDL (33.4–49.3 vs 60.4–68.4), fewer SUPRAL (9–11 vs 12–14), fewer INFRAL (10–11 vs 12–14), fewer RS (6–7 vs 8–9), fewer NCS (7–9 vs 13–16), fewer NR (2 vs 3–4), smaller GP (0–1 vs 4), the absence of tympanum scale, and the presence of occipital spines, which is absent in *A. murphyi*.

*Acanthosaura syrax* sp. nov. differs from *A. nataliae* in presenting a smaller SVL (62.5–101.2 vs 106.7–158.0), smaller TaL (72.4–106.9 vs 132.5–190.0), smaller SL (6.7–9.9 vs 12.0–19.9), smaller TD (1.9–3.2 vs 3.9–7.0), smaller PS/HL ratio (0.06–0.10 vs 0.36–0.52), smaller NSL/HL ratio (0.11–0.15 vs 0.58–0.75), smaller DS/HL ratio (0.05–0.09 vs 0.41–0.53), smaller FOREL (27.9–44.5 vs 58.4–85.0), smaller HINDL (33.4–49.3 vs 72.1–129.7), fewer VENT (56–60 vs 64–71), smaller RW (1.3–3.5 vs 4.6–6.1), smaller RH (0.7–1.5 vs 1.8–2.9), fewer NCS (7–9 vs 10–14), more NR (2 vs 1), fewer NSSLC (9–10 vs 13–16), smaller MW (0.7–1.8 vs 2.3–2.9), smaller MH (0.7–1.5 vs 2.0–2.9), smaller GP (0–1 vs 3–4), the absence of occipital spine, and the presence of black, diamond shaped, nuchal collar, and pale knee patches, which is absent in *A. nataliae*.

*Acanthosaura syrax* sp. nov. differs from *A. phongdienensis* in presenting a smaller TaL/SVL ratio (1.06–1.16 vs 1.50–1.90), greater HL (24.7–30.4 vs 18.6–23.8), greater HD (14.9–17.6 vs 10.4–13.6), and the presence of diastema, which is absent in *A. phongdienensis*.

*Acanthosaura syrax* sp. nov. differs from *A. prasina* Ananjeva, Ermakov, Nguyen, Nguyen, Murphy, Lukonina & Orlov, 2020 in presenting a smaller TaL/SVL ratio (1.06–1.16 vs 1.64–20.70), greater HL (24.7–30.4 vs 20.7–22.4), greater HW/HL ratio (0.21–0.25 vs 0.17–0.20), greater HW (14.9–17.6 vs 12.3–13.3), smaller ORBIT/HL ratio (0.08–0.11 vs 0.31–0.39), fewer TO (17–22 vs 23–26), and more CS (12–14 vs 5–6).

*Acanthosaura syrax* sp. nov. differs from *A. rubrilabris* in presenting a smaller TaL/SVL ratio (1.06–1.16 vs 1.31–1.88), smaller ORBIT/HL ratio (0.08–0.11 vs 0.28–0.35), greater TD/HL (0.16 vs 0.08–0.15), greater NS (7 vs 3–6), smaller NSW/NSL ratio (0.17–0.32 vs 0.43–0.89), greater FOREL/HINDL ratio (0.83–0.92 vs 0.67–0.78), and the absence of black eye patch.

*Acanthosaura syrax* sp. nov. differs from *A. titiwangsaensis* in presenting a smaller TaL (72.4–106.9 vs 136.0–174.0), greater HL (24.7–30.4 vs 20.0–24.3), smaller PS/HL ratio (0.06–0.10 vs 0.14–0.18), smaller NSW (0.4–1.3 vs 1.4–1.6), fewer DIASN (5–8 vs 10–13), fewer SUPRAL (9–11 vs 12–13), fewer TO (17–22 vs 23–27), smaller RW (1.3–3.5 vs 3.6–5.2), more RS (6–7 vs 5), fewer NS (5–7 vs 8), fewer NCS (7–9 vs 11–12), fewer NSCSL (6–8 vs 9–11), fewer NSSLC (9–10 vs 11–14), fewer PM (4 vs 5), smaller GP (0–1 vs 2–4), the absence of black eye patch, and the presence of pale knee patches, which is absent in *A. titiwangsaensis*.

*Acanthosaura syrax* sp. nov. differs from *A. tongbiguanensis* Liu & Rao, 2019 in presenting a smaller TaL/SVL ratio (1.06–1.16 vs 1.56–1.85), slightly smaller TD (1.9–3.2 vs 3.2–4.2), smaller PS/HL ratio (0.06–0.10 vs 0.13–0.19), slightly smaller NSL/HL ratio (0.11–0.15 vs 0.15–0.21), slightly smaller DS/HL ratio (0.05–0.09 vs 0.09–0.13), smaller HINDL (33.4–49.3 vs 54.1–63.9), fewer TO (17–22 vs 25–28), smaller OS/HL ratio (0.09–0.13 vs0.16–0.23), fewer NS (5–7 vs 8–9), fewer NCS (7–9 vs 10–13), and the absence of black eye patch.

## Discussion

The discovery of *Acanthosaura syrax* sp. nov. indicates that the species diversity of the genus *Acanthosaura* in Thailand has been significantly underestimated for a time. While most members of this genus have been described recently in other countries in Southeast Asia, particularly China and Vietnam, the ongoing discovery of new species, including this 23^rd^ recognized taxon, suggests that high-elevation mountain ranges still harbor substantial undocumented biodiversity (Nguyen et al. 2018, 2019; Liu and Rao 2019; Ananjeva et al. 2020; Liu et al. 2020, 2022; Le et al. 2025; Ngo et al. 2025). Historically, the taxonomic status of mountain-dwelling lizards in Southeast Asia was often obscured by the presence of cryptic species within complexes such as the *A. lepidogaster* complex (Thai National Parks 2013b). Restricted to the highlands of Mae Wong National Park, these populations previously thought to be a wider ranging species or closely related to *A. lepidogaster* are now recognized as new distinct lineages.

Morphologically, the unique characteristics of *A. syrax* sp. nov. further justify its distinct status. It is characterized by its small body size (SVL 62.4–78.5 mm) and possessing the smallest nuchal and dorsal scales recorded in the genus (NSL 2.4–4.7 mm, DS 1.0–2.6 mm). These traits suggest that *A. syrax* sp. nov. has evolved as a specialized high-altitude lineage, restricted primarily to the evergreen forests of the Mae Wong National Park Range at elevations above 1,300 m a.s.l.

The multi-locus molecular data generated in this study provides robust evidence supporting the status of *A. syrax* sp. nov. as a distinct evolutionary lineage. Analysis of the mitochondrial ND2 gene demonstrates that *A. syrax* sp. nov. exhibits high interspecific genetic divergence (0.14–0.21%) from all other analyzed Thai congeners. Most notably, a substantial genetic distance (0.14–0.16%) separates the new species from *A. lepidogaster*, its closest and morphologically most similar congener in western and northern Thailand. This high level of divergence is fully corroborated by the COI dataset, which shows deep interspecific distances between *A. syrax* sp. nov. and *A. lepidogaster* (16.64–17.53%). Furthermore, the new species maintains significant genetic isolation from other distributed congeners across China, Myanmar, and Vietnam, with COI pairwise distances ranging from 14.96% in *A. capra* to 28.13% in *A. grismeri*.

Beyond simple genetic distance thresholds, the robustness of our taxonomic conclusions is strongly reinforced by our updated phylogenetic framework. Both the Maximum Likelihood (ML) and Bayesian Inference (BI) analyses across both independent markers yielded congruent topologies (Figs 7, 8). In all reconstructions, *A. syrax* sp. nov. is recovered as a highly supported monophyletic lineage (UFB ≥ 95% and BPP ≥ 0.95) completely isolated from existing clades. This exceptional node support confirms that the new species is not a localized variant of an existing complex, but rather a distinct, independently evolving species. The significant genetic distances coupled with reciprocal monophyly firmly validate the formal description of *A. syrax* sp. nov.

The distribution of this species appears limited to high-altitude refugia, which are increasingly vulnerable to environmental shifts. While our field surveys indicate that populations in certain areas of Mae Wong National Park remain present, they are naturally fragmented by the rugged topography of the mountain range. This habitat fragmentation poses a significant long-term threat, as isolated populations may face difficulties in gene exchange, potentially leading to inbreeding and reduced genetic fitness. Such factors are detrimental to the survival of high-altitude specialists that cannot easily migrate to lower, warmer elevations. Despite our inability to locate *A. lepidogaster* within this specific high-altitude area, we encountered the species approximately 50 km to the north in Khlong Lan National Park. There, *A. lepidogaster* occurs in low-to-mid-elevation habitats characterized by significantly warmer conditions.

Surprisingly, *Acanthosaura syrax* sp. nov. shares a superficial resemblance to *A. lamnidentata* Boulenger, 1885 (currently a synonym of *A. lepidogaster*) from Pegu, Myanmar, specifically in the presence of small spines and a similar body plan. However, their morphological characteristics are clearly distinct: *A. syrax* sp. nov. exhibits significantly smaller proportions across all key diagnostic metrics when compared to *A. lamnidentata*, including SVL (62.5–101.2 vs 110), TAL (72.4–106.9 vs 170), FOREL (27.90–44.50 vs 57), and HINDL (33.40–49.30 vs 92) (Boulenger, 1885). Furthermore, most morphological characteristics of *A. lamnidentata* align more closely with the *A. lepidogaster* complex than with *A. syrax* sp. nov. Geographically, these populations are isolated by the formidable barrier of the Tenasserim Mountains, as well as by distinct ecological conditions associated with high-elevation habitats. These significant morphological and biogeographical differences provide strong evidence that these two populations represent distinct taxa.

Consequently, we suggest that the local government and National Park authorities strengthen the protection of these high-elevation habitats. Protecting the primary evergreen forest is the most effective strategy for ensuring the survival of *A. syrax* sp. nov. and other sympatric fauna. We recommend adopting this new species together with other threatened creatures [e.g. Asian forest tortoise (*Manouria emys* Schlegel & Müller, 1844), Oldham’s leaf turtle (*Cyclemys oldhamii* Gray, 1863), grey-sided thrush (*Turdus feae* (Salvadori, 1887)), rufous-necked hornbill (*Aceros nipalensis* (Hodgson, 1829)), and the Indochinese tiger (*Panthera tigris* (Linnaeus, 1758))] as flagship representatives for the Mae Wong region to draw scientific and public attention to the conservation of Thailand’s rapidly changing mountain ecosystems. Future studies should continue to explore neighboring mountain ranges to determine the full extent of this species’ distribution and identify further cryptic diversity that likely remains hidden within the northern and western highlands.

## Supplementary Material

XML Treatment for Acanthosaura

XML Treatment for Acanthosaura syrax

## Appendix 1

**Table A1.** GenBank accession numbers of ND2 and COI sequences used in this study.

**Taxon**	**Voucher**	**Locality**	**GenBank**		**Reference**
			**ND2**	**COI**	
**Ingroup**					
*Acanthosaura syrax* sp. nov.	THNHM31129	Mae Wong, Kampheang Phet, Thailand	PX738375	PZ164171	This study
*Acanthosaura syrax* sp. nov.	THNHM31130	Mae Wong, Kampheang Phet, Thailand	PX738377	PZ164172	This study
*Acanthosaura syrax* sp. nov.	THNHM31131	Mae Wong, Kampheang Phet, Thailand	PX738378	PZ164173	This study
*Acanthosaura syrax* sp. nov.	THNHM31132	Mae Wong, Kampheang Phet, Thailand	PX738371	PZ164174	This study
*Acanthosaura syrax* sp. nov.	THNHM31133	Mae Wong, Kampheang Phet, Thailand	PX738372	PZ164175	This study
*Acanthosaura syrax* sp. nov.	THNHM31134	Mae Wong, Kampheang Phet, Thailand	PX738373	PZ164176	This study
*Acanthosaura syrax* sp. nov.	THNHM31135	Mae Wong, Kampheang Phet, Thailand	PX738374	PZ164177	This study
*Acanthosaura syrax* sp. nov.	THNHM31136	Mae Wong, Kampheang Phet, Thailand	PX738376	PZ164178	This study
*Acanthosaura armata*	NSMT–H4595	Asia	AB266452	–	Okajima and Kumazawa (2010)
*Acanthosaura armata*	–	Asia	NC014175	–	Okajima and Kumazawa (2010)
*Acanthosaura aurantiacrista*	THNHM28064	Mae Sariang, Mae Hong Son, Thailand	MH777406	–	Trivalairat et al. (2020)
*Acanthosaura aurantiacrista*	QSMI1446	Sop Khong, Omkoi, Chiang Mai, Thailand	MK798128	–	Trivalairat et al. (2020)
*Acanthosaura aurantiacrista*	THNHM28521	Sop Khong, Omkoi, Chiang Mai, Thailand	MK798129	–	Trivalairat et al. (2020)
*Acanthosaura aurantiacrista*	THNHM28522	Sop Khong, Omkoi, Chiang Mai, Thailand	MK798130	–	Trivalairat et al. (2020)
*Acanthosaura aurantiacrista*	QSMI1447	Sop Khong, Omkoi, Chiang Mai, Thailand	MK798131	–	Trivalairat et al. (2020)
*Acanthosaura aurantiacrista*	QSMI1448	Sop Khong, Omkoi, Chiang Mai, Thailand	MK798132	–	Trivalairat et al. (2020)
*Acanthosaura aurantiacrista*	THNHM28523	Sop Khong, Omkoi, Chiang Mai, Thailand	MK798133	–	Trivalairat et al. (2020)
*Acanthosaura brachypoda*	ROM 38118	Sa Pa, Lao Cai, Vietnam	–	MK695182	Nguyen et al. (2019)
*Acanthosaura brachypoda*	ROM 38119	Sa Pa, Lao Cai, Vietnam	–	MK695183	Nguyen et al. (2019)
*Acanthosaura brachypoda*	ROM 38120	Sa Pa, Lao Cai, Vietnam	–	MK695184	Nguyen et al. (2019)
*Acanthosaura capra*	MVZ222130	Vietnam	AF128498	–	Macey et al. (1997a)
*Acanthosaura capra*	BGM 01	Bu Gia Map NP, Binh Phuoc (now Dong Nai), Vietnam	–	MK239022	Nguyen et al. (2018)
*Acanthosaura cardamomensis*	FMNH263225	Kampot, Cambodia	GU817397	–	Wood Jr et al. (2010)
*Acanthosaura cardamomensis*	FMNH263261	Kampot, Cambodia	GU817400	–	Wood Jr et al. (2010)
*Acanthosaura coronata*	KIZ47	Bu Gia Map NP, Binh Phuoc (now Dong Nai), Vietnam	–	MK695186	Nguyen et al. (2019)
*Acanthosaura coronata*	KIZ48	Bu Gia Map NP, Binh Phuoc (now Dong Nai), Vietnam	–	MK695187	Nguyen et al. (2019)
*Acanthosaura coronata*	KIZ49	Bu Gia Map NP, Binh Phuoc (now Dong Nai), Vietnam	–	MK695188	Nguyen et al. (2019)
*Acanthosaura coronata*	ROM 42240	Cat Tien NP, Dong Nai, Vietnam	–	MK695189	Nguyen et al. (2019)
*Acanthosaura coronata*	ROM 42241	Cat Tien NP, Dong Nai, Vietnam	–	MK695190	Nguyen et al. (2019)
*Acanthosaura crucigera*	QSMI1592	Muang, Tak, Thailand	MH777408	–	Trivalairat et al. (2022)
*Acanthosaura crucigera*	QSMI1593	Muang, Tak, Thailand	MH777403	–	Trivalairat et al. (2022)
*Acanthosaura crucigera*	THNHM28057	Muang, Tak, Thailand	MH777402	–	Trivalairat et al. (2022)
*Acanthosaura crucigera*	CAS229582	Kawthaung, Tanintharyi, Myanmar	GU817389	–	Wood Jr et al. (2010)
*Acanthosaura crucigera*	CUMZR2008.05.26.1	Petchaburi, Thailand	HM143889	–	Wood Jr et al. (2010)
*Acanthosaura crucigera*	USNM587487	Tanintharyi, Ma Noe Lone, Lenya, Myanmar	–	MT607982	Mulcahy et al. (2018)
*Acanthosaura crucigera*	USNM587488	Tanintharyi, Ma Noe Lone, Lenya, Myanmar	–	MT607980	Mulcahy et al. (2018)
*Acanthosaura crucigera*	USNM587489	Tanintharyi, Ma Noe Lone, Lenya, Myanmar	–	MT607981	Mulcahy et al. (2018)
*Acanthosaura cuongi*	IEBR R.5250	Phu Yen (now Dak Lak), Vietnam	–	PP669986	Ngo et al. (2025)
*Acanthosaura cuongi*	IEBR R.5257	Phu Yen (now Dak Lak), Vietnam	–	PP669987	Ngo et al. (2025)
*Acanthosaura cuongi*	IEBR R.5251	Khanh Hoa, Vietnam	–	PP669985	Ngo et al. (2025)
*Acanthosaura grismeri*	IEBR-R6353	Krong Pong forest, Dak Lak, Vietnam	–	PV646694	Le et al. (2025)
*Acanthosaura grismeri*	IEBR-R6354	Krong Pong forest, Dak Lak, Vietnam	–	PV646695	Le et al. (2025)
*Acanthosaura grismeri*	IEBR-R6357	Krong Pong forest, Dak Lak, Vietnam	–	PV646696	
*Acanthosaura lepidogaster*	MVZ224090	Vinh Thu, Vietnam	AF128499	–	Macey et al. (2000)
*Acanthosaura lepidogaster*	MD001	Hainan, China	KR092427	–	Yu et al. (2015)
*Acanthosaura lepidogaster*	ROM 30505	Na Hang NR, Tuyen Quang, Vietnam	–	MK695191	Nguyen et al. (2019)
*Acanthosaura lepidogaster*	ROM 30507	Na Hang NR, Tuyen Quang, Vietnam	–	MK695192	Nguyen et al. (2019)
*Acanthosaura lepidogaster*	ROM 30677	Quang Thanh, Cao Bang, Vietnam	–	MK695193	Nguyen et al. (2019)
*Acanthosaura lepidogaster*	ROM 30680	Na Hang NR, Tuyen Quang, Vietnam	–	MK695194	Nguyen et al. (2019)
*Acanthosaura lepidogaster*	ROM 30712	Tam Dao NP, Vinh Phuc (now Phu Tho), Vietnam	–	MK695195	Nguyen et al. (2019)
*Acanthosaura liui*	KIZL2020003	Jianshui, Honghe, Yunnan, China	–	MT769763	Liu et al. (2020)
*Acanthosaura liui*	KIZL2020004	Jianshui, Honghe, Yunnan, China	–	MT769764	Liu et al. (2020)
*Acanthosaura liui*	KIZL2020005	Jianshui, Honghe, Yunnan, China	–	MT769765	Liu et al. (2020)
*Acanthosaura liui*	KIZL2020006	Jianshui, Honghe, Yunnan, China	–	MT769766	Liu et al. (2020)
*Acanthosaura liui*	KIZL2020007	Jianshui, Honghe, Yunnan, China	–	MT769767	Liu et al. (2020)
*Acanthosaura longicaudata*	KIZ_L0051	Jiangcheng, Yunnan, China	–	ON207528	Liu et al. (2022)
*Acanthosaura longicaudata*	KIZ_L0132	Jiangcheng, Yunnan, China	–	ON207529	Liu et al. (2022)
*Acanthosaura longicaudata*	KIZ_L0133	Jiangcheng, Yunnan, China	–	ON207530	Liu et al. (2022)
*Acanthosaura longicaudata*	KIZ_L0150	Jiangcheng, Yunnan, China	–	ON207531	Liu et al. (2022)
*Acanthosaura meridiona*	QSMI1594	Na Yong, Trang, Thaiand	MH777404	–	Trivalairat et al. (2022)
*Acanthosaura meridiona*	THNHM28061	Na Yong, Trang, Thaiand	MH777407	–	Trivalairat et al. (2022)
*Acanthosaura meridiona*	THNHM28062	Na Yong, Trang, Thaiand	MH777405	–	Trivalairat et al. (2022)
*Acanthosaura murphyi*	ITBCZ 4603	Deo Ca Forest, Phu Yen (now Dak Lak), Vietnam	–	MK239025	Nguyen et al. (2018)
*Acanthosaura murphyi*	ITBCZ 3533	Hon Ba NR, Khanh Hoa, Vietnam	–	MK239026	Nguyen et al. (2018)
*Acanthosaura nataliae*	ROM30629	Tram Lap, Gia Lai, Vietnam	–	MK695202	Nguyen et al. (2019)
*Acanthosaura nataliae*	ROM30631	Tram Lap, Gia Lai, Vietnam	–	MK695203	Nguyen et al. (2019)
*Acanthosaura nataliae*	ROM30632	Tram Lap, Gia Lai, Vietnam	–	MK695204	Nguyen et al. (2019)
*Acanthosaura phongdienensis*	ITBCZ6831	Phong Dien, Thua Thien Hue, Vietnam	–	MK695205	Nguyen et al. (2019)
*Acanthosaura phongdienensis*	ROM48013	Phong Dien, Thua Thien Hue, Vietnam	–	MK695206	Nguyen et al. (2019)
*Acanthosaura phongdienensis*	KIZ10657	Phong Dien, Thua Thien Hue, Vietnam	–	MK695207	Nguyen et al. (2019)
*Acanthosaura phongdienensis*	ITBCZ6830	Phong Dien, Thua Thien Hue, Vietnam	–	MK695208	Nguyen et al. (2019)
*Acanthosaura phongdienensis*	KIZ10695	Phong Dien, Thua Thien Hue, Vietnam	–	MK695209	Nguyen et al. (2019)
*Acanthosaura phongdienensis*	KIZ10697	Phong Dien, Thua Thien Hue, Vietnam	–	MK695210	Nguyen et al. (2019)
*Acanthosaura rubrilabris*	KIZ_L0057	Menghai, Yunnan, China	–	ON207534	Liu et al. (2022)
*Acanthosaura rubrilabris*	KIZ_L0010	Menghai, Yunnan, China	–	ON207535	Liu et al. (2022)
*Acanthosaura rubrilabris*	KIZ_L0006	Jinghong, Yunnan, China	–	ON207536	Liu et al. (2022)
*Acanthosaura rubrilabris*	KIZ_L0040	Jinghong, Yunnan, China	–	ON207537	Liu et al. (2022)
*Acanthosaura rubrilabris*	KIZ_L0137	Lancang, Yunnan, China	–	ON207543	Liu et al. (2022)
*Acanthosaura rubrilabris*	KIZ_L0140	Lancang, Yunnan, China	–	ON207544	Liu et al. (2022)
**Outgroup**					
*Calotes emma*	CAS223062	Rakhine State, Myanmar	DQ289460	–	Zug et al. (2006)
*Calotes emma*	KIZ1120	Ta Kou NR, Binh Thuan (now Lam Dong), VN	–	MK695211	Nguyen et al. (2019)

## Additional information

### Artificial Intelligence (AI) use

The authors accept full responsibility for the content of the manuscript, including the disclosure of any use of AI.

No AI tools were used in the preparation of this manuscript.

### Author ORCIDs

P. Trivalairat https://orcid.org/0000-0001-8849-8517

K. Trivalairat https://orcid.org/0000-0003-0005-0801

B. Lorsunyaluck https://orcid.org/0000-0002-0300-5937

### Data availability

All of the data that support the findings of this study are available in the main text or Supplementary Information.

## Supplementary materials

Supplementary material 1 Supplementary tables Data type xlsx Explanation note **table SS1**. The comparisons of the morphometric measurements and meristic data for all currently recognised species in comparison with *A. syrax* sp. nov. and other recognised *Acanthosaura* species. **table S2**. Morphological comparison of nuchal and dorsal crest heights among *Acanthosaura* species. https://binary.pensoft.net/file/1731697This dataset is made available under the Open Database License (http://opendatacommons.org/licenses/odbl/1.0/). The Open Database License (ODbL) is a license agreement intended to allow users to freely share, modify, and use this Dataset while maintaining this same freedom for others, provided that the original source and author(s) are credited. Poramad Trivalairat, Krittiya Trivalairat, Nawee Moungkhaek, Simon Caponov, Benchapol Lorsunyaluck
